# AAV-mediated *in vivo* functional selection of tissue-protective factors against ischaemia

**DOI:** 10.1038/ncomms8388

**Published:** 2015-06-11

**Authors:** Giulia Ruozi, Francesca Bortolotti, Antonella Falcione, Matteo Dal Ferro, Laura Ukovich, Antero Macedo, Lorena Zentilin, Nicoletta Filigheddu, Gianluca Gortan Cappellari, Giovanna Baldini, Marina Zweyer, Rocco Barazzoni, Andrea Graziani, Serena Zacchigna, Mauro Giacca

**Affiliations:** 1 Molecular Medicine Laboratory, International Centre for Genetic Engineering and Biotechnology (ICGEB), 34149 Trieste, Italy; 2 Department of Medical, Surgical and Health Sciences, University of Trieste, 34127 Trieste, Italy; 3 Department of Translational Medicine, University of Piemonte Orientale ‘A. Avogadro’, 28100 Novara, Italy

## Abstract

Functional screening of expression libraries *in vivo* would offer the possibility of identifying novel biotherapeutics without a priori knowledge of their biochemical function. Here we describe a procedure for the functional selection of tissue-protective factors based on the *in vivo* delivery of arrayed cDNA libraries from the mouse secretome using adeno-associated virus (AAV) vectors. Application of this technique, which we call FunSel, in the context of acute ischaemia, revealed that the peptide ghrelin protects skeletal muscle and heart from ischaemic damage. When delivered to the heart using an AAV9 vector, ghrelin markedly reduces infarct size and preserves cardiac function over time. This protective activity associates with the capacity of ghrelin to sustain autophagy and remove dysfunctional mitochondria after myocardial infarction. Our findings describe an innovative tool to identify biological therapeutics and reveal a novel role of ghrelin as an inducer of myoprotective autophagy.

## Abstract (web summary)

Cell-based screening assays allow functional testing of chemicals but do not mimic the *in vivo* situation well. Here, the authors report a method for the discovery of secreted cytoprotective factors in mice and use it to demonstrate that the hormone ghrelin protects cardiac muscle from ischaemic damage.

## Graphical abstract

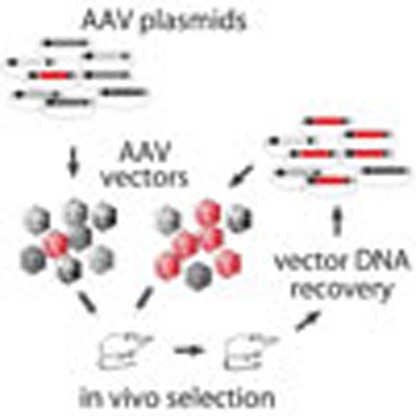

Since the birth of genetic engineering, the screening of genetic libraries has provided a powerful means for the identification of genes, proteins and small nucleic acids possessing desirable characteristics. Initial screenings, based on nucleic acid hybridization or antibody reactivity in cell culture-based assays, were later complemented by functional screenings, relying on selection for survival, transformation or modulation of cell function. Although these cell-based approaches have generated essential biological information with important upshots on drug target identification, they suffer a major, unavoidable limitation, namely, they cannot predict the *in vivo* relevance of the selected molecules. In addition, several factors exert beneficial function *in vivo* by multiple, pleiotropic mechanisms, the individual relevance of which cannot be anticipated by *in vitro* assays. Thus, in only a very few cases, the factors that emerged from these *in vitro* screenings have advanced to clinical use. In contrast, the possibility of screening libraries for a given function directly *in vivo* would allow for the direct identification of factors with proven functional relevance.

Here, we report on the development of an *in vivo*, functional selection procedure (FunSel) for the identification of factors protecting against tissue degeneration, and we provide proof-of-principle application of this procedure to identify therapeutic factors for acute myocardial infarction (MI) and limb ischaemia. We generated an arrayed library of cDNAs encoding murine-secreted factors in vectors based on the adeno-associated virus (AAV); then, we submitted this library to iterative cycles of selection in the ischaemic skeletal muscle to identify factors with myoprotective function; finally, we tested and characterized the effect of the selected molecule in models of peripheral and cardiac ischaemia. FunSel exploits three unique properties of AAV vectors, namely their exquisite *in vivo* tropism for post-mitotic cells (including cardiomyocytes and skeletal muscle fibres^1^,^2^), their prolonged transgene expression and the possibility to produce high titre vector preparations, often in excess of 1 × 10^14^ viral particles per ml (recently reviewed in ref. 3).

Under our experimental conditions, FunSel led to the selection of ghrelin, a 28-amino-acid peptide hormone, which is normally produced by the stomach and octanoylated on serine 3 by the ghrelin-O-acyltransferase (GOAT) enzyme^4^,^5^ to generate acyl ghrelin. This modified peptide binds the growth hormone secretagogue receptor-1a (GHSR-1a) to induce growth hormone release and perform multiple endocrine functions^6^,^7^. The major form of circulating ghrelin, however, is not acylated (des-acyl ghrelin) and acts through a still unidentified receptor, different from GHSR-1a^8^,^9^,^10^. The mechanism by which des-acyl ghrelin exerts its functions in various tissues still remains largely unknown.

Autophagy has progressively gained remarkable attention over the last few years as an adaptive response against stress, capable to protect various tissues from injury and maintain tissue homeostasis by the removal of dysfunctional organelles through the lysosomal degradative pathway^11^,^12^,^13^,^14^,^15^,^16^. In the heart, converging evidence has indicated that autophagy exerts beneficial effects^17^. During heart failure, this process protects against progressive cardiac dysfunction^17^; ischaemia itself stimulates autophagy as an adaptive mechanism^18^. Consistent with this protective role, defects in cardiomyocyte autophagy induce cardiac dysfunction in both animal models^19^ and patients with inherited defects of proteins involved in the autophagic process^20^.

We report here that des-acyl ghrelin, once expressed in the heart after MI and independent from its endocrine function, remarkably, protects cardiac cells from ischaemic damage and sustains cardiac function over time, whereas, in the skeletal muscle, it promotes muscle fibre regeneration after ischaemia. These effects parallel the previously uncovered property of unmodified ghrelin to directly induce cardiomyocyte and skeletal muscle fibre autophagy, resulting in the removal of dysfunctional mitochondria.

## Results

### *In vivo* functional selection of tissue protective factors

**Figure 1:**
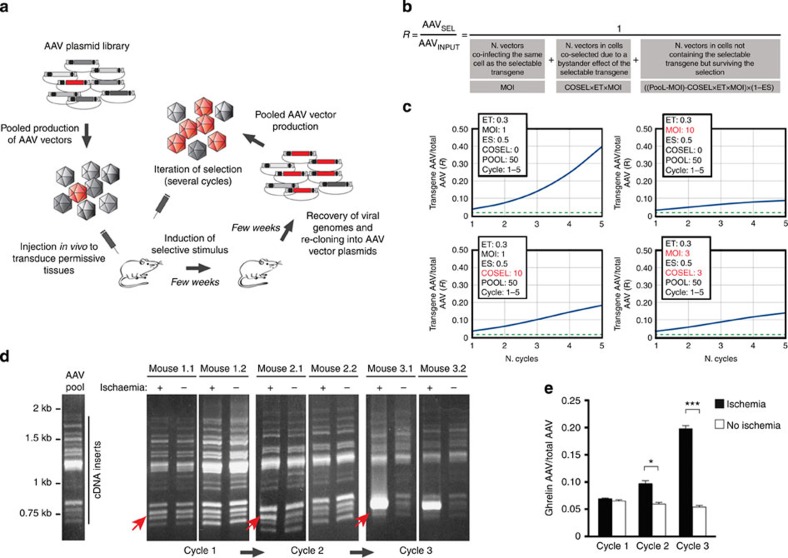
Identification of ghrelin as a myoprotective factor by the application of FunSel. (**a**) Outline of the FunSel procedure. Red: selected gene. (**b**) Formula defining the efficiency of transgene selection (*R*) according to the variables affecting FunSel (Supplementary Table 1). (**c**) FunSel simulation. In an ideal situation, the number of transduced cells is set at 30% (ET=0.3, as it is common for muscle and cardiac AAV transduction^3^), each vector enters one cell (MOI=1), there is no bystander effect (COSEL=0), and 50% of cells with the transgene are selected (ES=0.5). The upper left panel shows the effect of performing five iterative cycles of selection under these conditions with a pool of 50 vectors (POOL=50). The frequency of a selected vector, starting from 0.02 (1 50^−1^) before the selection, doubles after one cycle and reaches 0.14 and 0.40 after 3 and 5 cycles, respectively. Increasing the MOI or COSEL effect decrease efficiency of selection, as shown in the lower left (MOI=10) and upper right (COSEL=10) panels, respectively. Under these conditions, three cycles of selection enrich the desired transgene 2.3- or 5-folds, respectively. In a realistic scenario, transduction occurs with 50 vectors (POOL=50) at an efficiency of 30% (ET=0.3), three vectors per cell (MOI=3), two selected cells in addition to the transduced one (COSEL=3) and ES=0.5. Under these conditions, enrichment for the transgene becomes 2-fold after one cycle, 4.5-fold after three cycles and 7-fold after five cycles (lower right panel). (**d**) Iterative enrichment for the ghrelin cDNA after three cycles of selection in ischaemic skeletal muscles. The pictures shows PCR fragments corresponding to the cDNA inserts, generated with primers flanking the AAV inserts. The first ladder corresponds to vector DNAs before injection. The subsequent ladders correspond to DNAs extracted from ischaemic and non-ischaemic legs of two representative animals at each cycle of selection. An arrow indicates the ghrelin band. (**e**) Real-time PCR quantification of the relative amount of the ghrelin cDNA over the total AAV DNA at each cycle of selection. Bars represent mean±s.e.m. (*n*=12 per group per cycle). Student’s *t-*test; *P<0.05, **P<0.01.

FunSel is based on iterative cycles of *in vivo* transduction of an AAV vector library coding for different transgenes, followed by the induction of a selective stimulus (Fig. 1a). A library of AAV plasmids, each one coding for a specific factor, is used for the batch production of AAV vectors, which are then injected *in vivo* to transduce permissive cells (for example, myofibers, cardiomyocytes, photoreceptors, pancreas β-cells); in principle, each vector transduces a different cell. After a few weeks, a selective stimulus is applied (for example, ischaemia for skeletal muscle or heart, retinal degeneration or chemical induction of cell death). Then, vector cDNA inserts are recovered from the injected tissue and used to generate a new AAV preparation, which is used for another cycle of selection. Should any of the viral transgenes exert a protective or regenerative effect on the cell expressing it (shown in red in Fig. 1a), it becomes progressively enriched along the iterative cycles of selection.

Theoretical considerations were elaborated to support the feasibility of FunSel. The ultimate purpose of the procedure is the *in vivo* selection of a specific AAV vector, coding for a desired transgene (*T*) from a pool of several AAVs (*A*), each one coding for a different factor. The ratio between T and A (herein defined as *R*=*T A*^−1^ (equation 1)) indicates the success of the selection procedure. At least six parameters influence AAV vector-mediated selection: (i) the initial number of different vectors included in the original pool (POOL), (ii) the efficiency of transduction (ET), (iii) the number of different vectors entering each cell (multiplicity of infection, MOI), (iv) the efficiency of selection (ES, measuring the selective advantage offered by the transgene), (v) the effect of a selected transgene on neighbouring, non-transduced cells (COSEL) and (vi) the number of iterative selection cycles (CYCLE; Supplementary Table 1). At each cycle of selection, when the number of vectors with the desired transgene is set to 1 (*T*=1), the total number of AAV vectors (*A*) after transduction and selection consists of the sum of: (i) the number of vectors co-infecting the same cell as the desired vector (MOI); plus (ii) the number of vectors present in the cell close to the selected cells and co-selected with this (COSEL × ET × MOI); plus (iii) the total number of vectors in the cells containing other transgenes but surviving the selection ((POOL−MOI)−COSEL × ET × MOI) × (1−ES). Thus, at the end of the FunSel procedure, *R* becomes as shown in Fig. 1b, to be compared with its value at start, when *R*=1 POOL^−1^ (equation 2).

Figure 1c shows how varying MOI and COSEL affect selection efficiency. In all of the cases considered, robust transgene selection (over tenfold) is achieved with a relatively small number of selection cycles (from 1 to 5). These mathematical considerations support the feasibility of FunSel over a variegated range of experimental settings.

### Virologic feasibility of FunSel

We generated a collection of 100 sequence-verified, AAV vectors coding for secreted proteins, as identified in the FANTOM3 project^21^. These genes were representative of various categories, including cytokines and interleukins (11 clones), hormones and growth factors (25), extracellular matrix proteins (11), secreted enzymes (18), other proteins of miscellaneous function (22) and proteins of unknown function (13; cf. complete list in Supplementary Table 2). The cDNA of all these factors was cloned into an AAV vector plasmid under the control of the CMV IE promoter.

To explore the efficiency of simultaneous packaging of multiple AAV vector plasmids, we obtained AAV2 pools with complexities of 25, 50 and 100 different vectors (Supplementary Table 2). By analysing the frequency of ten randomly chosen inserts in the three preparations, we found that cDNA inserts of up to 1,500 bp were packaged at an efficiency within ±2-fold compared with those expected, and those with a longer size, with an efficiency within ±3-fold, irrespective of pool complexity (Supplementary Fig. 1a). Both intervals appeared acceptable for the purpose of FunSel.

To verify the infectivity of individual AAV vectors within their pools, we transduced skeletal muscles with the AAV2 pools composed of either 25 or 100 clones; in both cases, the relative abundance of each of the individual constructs at 3 weeks after transduction was in the ±2-fold range compared with the value expected according their dilution in the infecting pool (Supplementary Fig. 1b). Finally, we ascertained the expression of the transduced clones *in vivo*. The same number of viral particles of the AAV2 pools composed of 25, 50 or 100 vectors, as well as two individual AAV2 vectors coding for two specific clones in the pools (clones E2 and C12), were injected into the skeletal muscle. After 3 weeks in all cases, the relative abundance of E2 and C12 mRNAs corresponded to the dilution of the encoding vectors in the original mixture (Supplementary Fig. 1c,d). Vector pool complexities of 25 and 50 allowed relatively robust expression of the investigated transgenes.

### Functional selection of ghrelin after hindlimb ischaemia

We wanted to obtain a proof-of-principle of FunSel in identifying factors protecting against acute ischaemia. We bilaterally injected a pool of 30 different AAV2 vectors (coding for the factors listed in Supplementary Table 2) into the tibialis anterior muscles of CD1 mice (1 × 10^10^ viral genomes (vg) per limb); 2 weeks after injection, the left femoral artery was resected to induce unilateral ischaemia. After 2 additional weeks, the vector cDNAs from both the ischaemic and non-ischaemic leg were recovered by PCR using a primer pair flanking the cDNA inserts, pooled according to treatment and re-cloned into AAV vector plasmids to start an additional selection cycle. Selection was applied for three iterative cycles (*n*=12 per group per pool per cycle).

The PCR ladders obtained from the input viral lysate and from the DNAs recovered from the legs of two representative animals at each selection cycle are shown in Fig. 1d. The procedure led to the progressive enrichment of a single cDNA species (quantification in Fig. 1e). DNA sequencing identified it as the cDNA coding for the neuroendocrine peptide ghrelin. None of the other 70 genes considered in original group of 100, which were analysed in pools of 35, were significantly enriched compared with ghrelin (not shown).

### Ghrelin improves muscle recovery after hindlimb ischaemia

**Figure 2:**
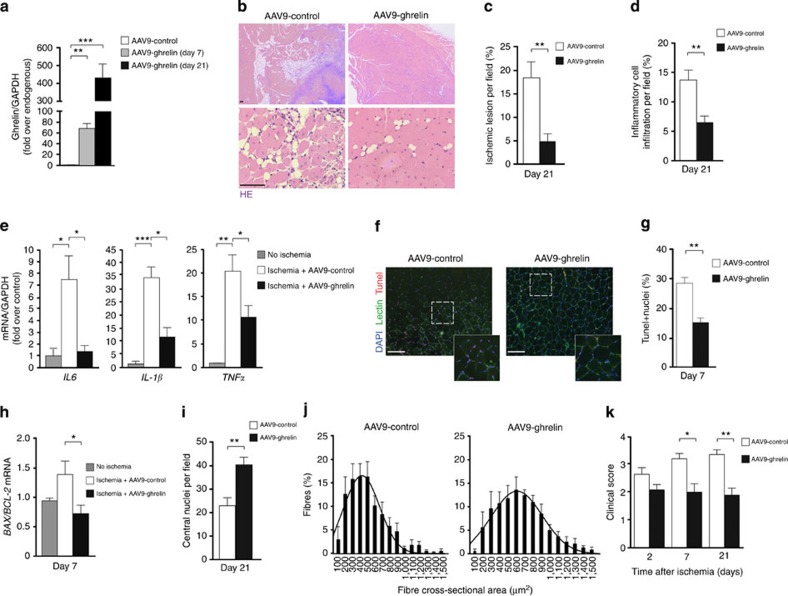
Ghrelin improves muscle functional recovery after femoral artery resection. (**a**) Real-time PCR quantification of ghrelin mRNA in tibialis anterior muscles transduced with 1 × 10^11^ vg of AAV. Values are normalized for glyceraldehyde 3-phosphate dehydrogenase (GAPDH) and expressed as fold over endogenous (*n*=5). (**b**) Haematoxylin-eosin (HE) staining of tibialis anterior muscles at day 21 after femoral artery resection. Scale bar, 50 μm. (**c**) Quantification of ischaemic lesion in muscles injected with AAV9-ghrelin. Values are expressed as percentage per field (*n*=7 per group, four fields counted for each animal). (**d**) Quantification of inflammatory cell infiltration. Values are expressed as percentage of cells per field (*n*=7 per group, four fields per animal). (**e**) Expression levels of inflammatory cytokines, analysed by real-time PCR, at day 7 after transduction and surgery. Values are normalized for GAPDH and expressed as fold over untreated (*n*=5). (**f**,**g**) Analysis of apoptosis by TUNEL. Representative images (**f**) and quantification (**g**) of TUNEL-positive nuclei in the muscle areas adjacent to the lesions (*n*=5). Red: apoptotic nuclei; blue: 4′-6-diamidino-2-phenylindole (DAPI); green: lectin-stained muscle fibres. Scale bar, 100 μm. (**h**) Ratio between *BAX* and *BCL-2* mRNAs in AAV9-injected muscles, 7 days after femoral artery resection. Values are normalized for GAPDH and expressed as fold over untreated (*n*=5). (**i**) Number of fibres with central nuclei per field as a marker of muscle regeneration at 21 days after treatment (*n*=7 per group, four fields per animal). (**j**) Fibre size analysis after ischaemia and AAV9 injection. The histograms show the distribution of the fibre cross-sectional areas (μm^2^); a normal distribution curve is superimposed. Analysis of 20 cross-sections from six animals per group was performed. *P*<0.01. (**k**) Limb function analysed at days 2, 7 and 21 after treatment by assigning a clinical score, according to established criteria (*n*=25 per group)^60^. All values are mean±s.e.m. Pairwise comparison was performed with the Student’s *t*-test (**c**,**d**,**g**,**i**); one-way analysis of variance (ANOVA) and Bonferroni/Dunn’s *post hoc* test were used to compare multiple groups (**a**,**e**,**h**); two-way ANOVA was used in **k**; the Kolmogorov–Smirnov test was used to compare distributions in **j**. *P<0.05, **P<0.01, ***P<0.001 relative to control.

We verified that AAV9-ghrelin exerted a direct protective effect after ischaemic muscle injury, in agreement with previous findings using the ghrelin peptide^22^. Injection of AAV9-ghrelin (1 × 10^11^ vg) in the tibialis anterior muscle of CD1 mice immediately after femoral artery resection led to robust transgene expression over time (Fig. 2a), which prevented fibre degeneration (Fig. 2b), reduced size of the ischaemic lesion, inflammatory cell infiltration and expression of several inflammatory cytokines (Fig. 2c,d,e respectively). Preservation of muscle mass correlated with marked protection from apoptosis (Fig. 2f–h) and improved muscle regeneration, indicated by the significantly increased number of centronucleated fibres (Fig. 2i) and the right-skewed distribution of the fibre cross-sectional area (Fig. 2j), eventually resulting in improved limb function in ghrelin-expressing muscles (Fig. 2k). The myoprotective effect of ghrelin correlated with increased numbers of both capillaries and arterioles, likely as a consequence of tissue survival and regeneration (Supplementary Fig. 2c).

### Ghrelin preserves cardiac function after MI

**Figure 3:**
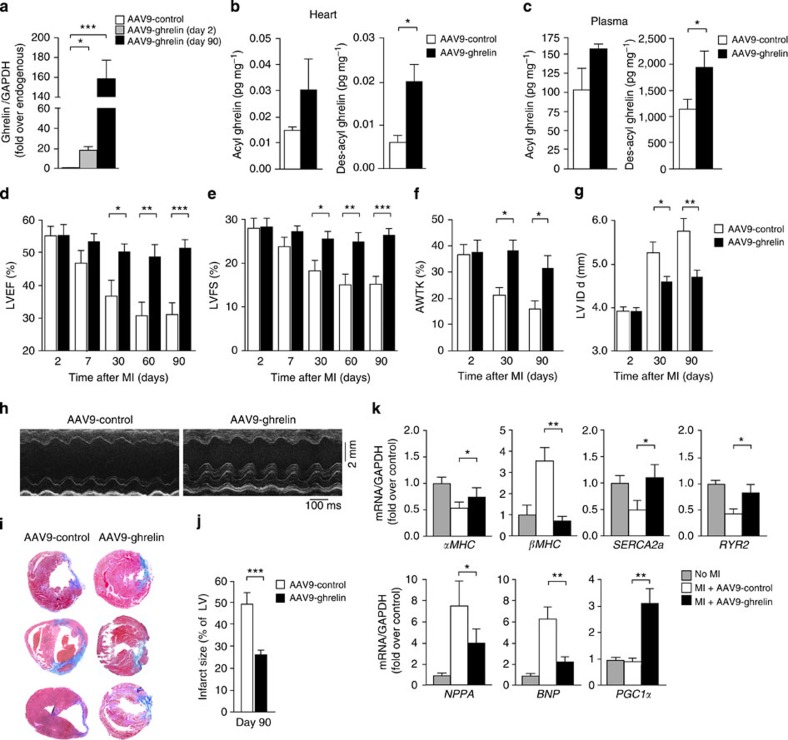
Ghrelin preserves cardiac function and reduces infarct size after myocardial infarction (MI). (**a**) Real-time PCR quantification of ghrelin mRNA in total ventricular RNA extracted 2 or 90 days after AAV9 injection and MI induction. Values are normalized for glyceraldehyde 3-phosphate dehydrogenase (GAPDH) and expressed as fold over endogenous (*n*=5). (**b**,**c**) Intracardiac (**b**) and plasmatic (**c**) levels of acyl and des-acyl ghrelin evaluated by EIA 90 days after intracardiac vector injection (*n*=4). (**d**–**g**) Echocardiographic analysis in AAV9-injected and control mice at 2, 7, 30, 60 and 90 days after MI. Left ventricular ejection fraction (LVEF; **d**), left ventricular fractional shortening (LVFS; **e**), anterior wall thickening (AWTK; **f**) and diastolic LV internal diameter (LVID d; **g**) of infarcted hearts treated either with AAV9-ghrelin or AAV9-control were measured (*n*=12 per group). **(h**) Representative M-mode echocardiographic images 90 days after AAV9-control or AAV9-ghrelin injection and MI induction. (**i**) Representative images of whole transverse sections after Azan-trichromic staining of hearts transduced with AAV9-control or AAV9-ghrelin. Fibrotic areas are stained in blue. (**j**) Quantification of infarct size expressed as percentage of LV (*n*=12 per group). (**k**) Cardiac expression levels of the indicated genes in AAV9-control and AAV9-ghrelin-treated hearts, 90 days after MI. Values are normalized for GAPDH and expressed as fold over untreated (*n*=6). All values are mean±s.e.m. Pairwise comparison was performed with the Student’s *t*-test (**b**,**c**,**j**); one-way analysis of variance (ANOVA) and Bonferroni/Dunn’s *post hoc* test were used to compare multiple groups (**a**,**k**); two-way ANOVA was used in **d**–**g**. *P<0.05, **P<0.01, ***P<0.001 relative to control.

Next, we wanted to assess whether AAV9-ghrelin protected the heart after MI. Adult CD1 mice (*n*=25 per group) underwent permanent left descendent coronary artery ligation and were immediately injected with 1 × 10^11^ viral particles of AAV9-ghrelin or of a control AAV9 vector, in the peri-infarcted area. Ghrelin mRNA was found overexpressed in the heart, already at day 2 after vector injection (∼20-fold), and remained high for at least 3 months (160-fold; Fig. 3a); at 15 days, AAV-ghrelin genomes and transgene expression were also observed in the liver (Supplementary Fig. 3a,b, respectively). Consistently, both intracardiac and plasmatic levels of des-acyl ghrelin were significantly increased in the AAV9-ghrelin-injected animals (Fig. 3b,c, respectively).

As evaluated by echocardiography at 2, 7, 30, 60 and 90 days post MI, both the left ventricle (LV) ejection fraction (Fig. 3d) and the LV fractional shortening (Fig. 3e) were significantly preserved in the infarcted mice injected with AAV9-ghrelin. These parameters were maintained over time (at day 90 post MI, LV ejection fraction: 30.8±3.4% versus 50.7±2.7%; LV fractional shortening: 14.9±1.8% versus 26.0±1.6% in control and ghrelin-transduced animals, respectively). At both 1 and 3 months after cardiac gene transfer, the LV end-systolic anterior wall thickening (Fig. 3f) of the infarcted hearts injected with AAV9-ghrelin was also markedly improved (20.7±2.8% versus 37.1±5.4 and 15.4±3.3% versus 30.8±4.5% in control and ghrelin-transduced animals, respectively at day 30 and 90 after infarction). Finally, the diastolic LV internal diameter (Fig. 3g) was significantly larger in the control animals at both 1 and 3 months after MI. Representative M-mode echocardiograms at the latter time are shown in Fig. 3h.

Morphometric analysis on trichromic-stained heart sections at day 90 indicated that AAV9-ghrelin-treated mice showed significant preservation of LV-contractile tissue and reduction of the fibrotic area (infarct size: 51.3±4.8% versus 27±1.9% of the LV in control and ghrelin-transduced animals, respectively; Fig. 3i,j).

Consistent with echocardiographic and morphometric observations, AAV9-ghrelin significantly counteracted the characteristic pattern of gene expression typically associated with pathological LV remodelling, consisting in the overexpression of *α-MHC* and cardiac natriuretic peptides (*BNP* and *NPPA*), and in decreased levels *of β-MHC, SERCA2a, RYR2* and *PGC1α* (Fig. 3k).

Finally, at day 7, post MI, when inflammatory cell massively infiltrated the heart of control mice, ghrelin significantly blunted this effect (*n*=5 per group; Supplementary Fig. 4a,b), with a parallel decrease in the levels of the inflammatory cytokines *IL-1β, IL6* and *TNFα* (Supplementary Fig. 4c).

Collectively, these results indicate that ghrelin is cardioprotective after MI, reducing infarct size and preserving myocardial viability and function.

### Ghrelin protects myocardial cells from apoptosis

**Figure 4:**
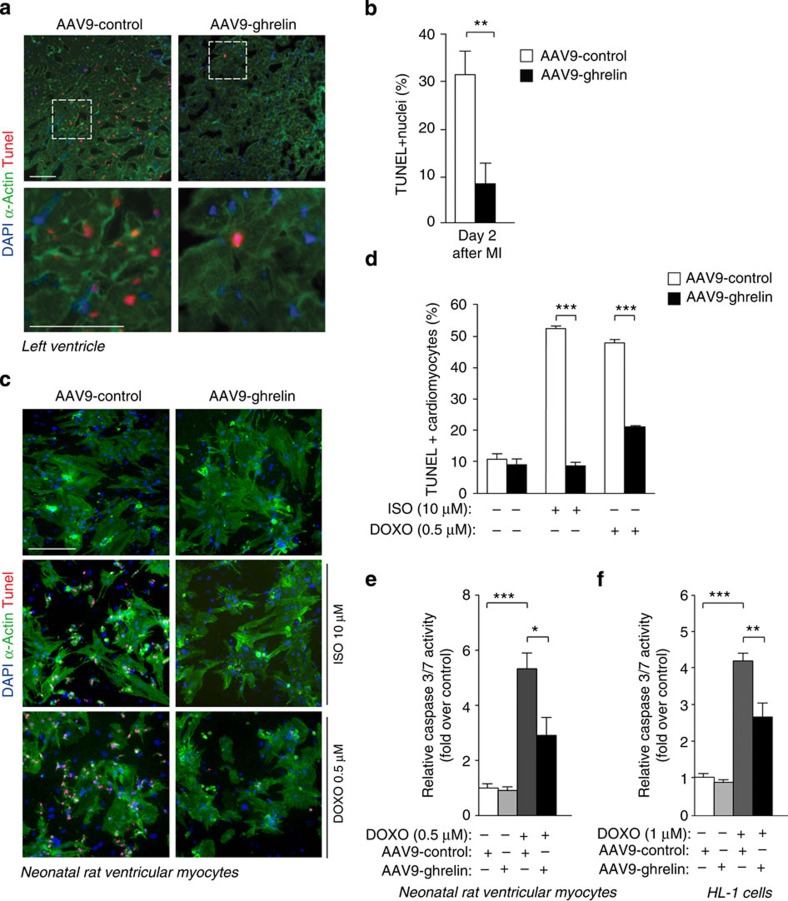
AAV9-ghrelin exerts anti-apoptotic effect on cardiomyocytes exposed to toxic or ischaemic damage *in vitro* and *in vivo*. (**a**) TUNEL staining of the infarct border zone in hearts injected with AAV9-control or AAV9-ghrelin 2 days after MI. Nuclei are stained blue with 4′-6-diamidino-2-phenylindole (DAPI) and cardiomyocytes green by an antibody against α-actinin. Red nuclei indicate apoptotic cells. Scale bar, 100 μm. (**b**) Quantification of TUNEL-positive nuclei (% of total) in the peri-infarctual region of AAV9-control and AAV9-ghrelin-treated mice (*n*=8 per group). (**c**) Rat neonatal cardiomyocytes, transduced with AAV9-control or AAV9-ghrelin (MOI=5 × 10^4^ vg per cell, transduction efficiency >40%) were either left untreated or treated with (−)-Isoproterenol hydrochloride 10 μM (ISO) or doxorubicin hydrochloride 0.5 μM (DOXO); after 24 or 48 h, respectively, cells were fixed and stained with TUNEL assay. Nuclei are stained blue with DAPI and cardiomyocytes green by an antibody against α-actinin. Red nuclei indicate apoptotic cells. Scale bar, 100 μm. (**d**) Quantification of cardiomyocytes TUNEL-positive nuclei (% of total) after transduction with AAV9-control or AAV9-ghrelin and (−)-Isoproterenol or doxorubicin treatment. Quantification was performed using the ImaJ software (*n*=4). (**e**,**f**) Caspase 3/7 activation analysis in rat neonatal cardiomyocytes (**e**) and HL-1 cells (**f**) transduced with AAV9-ghrelin or AAV9-control and treated with doxorubicin 0.5 μM and 1 μM, respectively, for 20 h (*n*=12). All values are mean±s.e.m. Pairwise comparison was performed with the Student’s *t*-test (**b**,**d**); one-way analysis of variance and Bonferroni/Dunn’s *post hoc* test were used to compare multiple groups (**e**,**f**). **P<0.01, ***P<0.001 relative to control.

The preservation of cardiac tissue observed in hearts injected with AAV9-ghrelin following MI suggested that the factor, similar to what had been observed in the skeletal muscle, might protect cardiomyocytes from ischaemia-induced apoptosis. Indeed, the number of apoptotic nuclei in the animals injected with this vector at day 2 after MI was markedly decreased (*n*=8 per group; 30.7±5.4% versus 8.3±4.6% TUNEL (TdT-mediated dUTP nick-end labelling)-positive cells in control and ghrelin-transduced animals, respectively; representative images and quantification in Fig. 4a,b, respectively).

A similar protective effect of AAV9-ghrelin vector (MOI: 5 × 10^4^ vg per cell) was also observed in neonatal rat ventricular cardiomyocytes treated with the cardiotoxic agents isoproterenol or doxorubicin, by quantifying the number of TUNEL-positive nuclei (Fig. 4c,d). The *in vitro* antiapoptotic effect of AAV9-ghrelin was confirmed by a reduction in caspase 3/7 activation in both neonatal cardiomyocytes and HL-1 cells, after 20 h of doxorubicin treatment (0.5 and 1 μM, respectively; Fig. 4e,f).

### Ghrelin stimulates autophagy after MI

**Figure 5:**
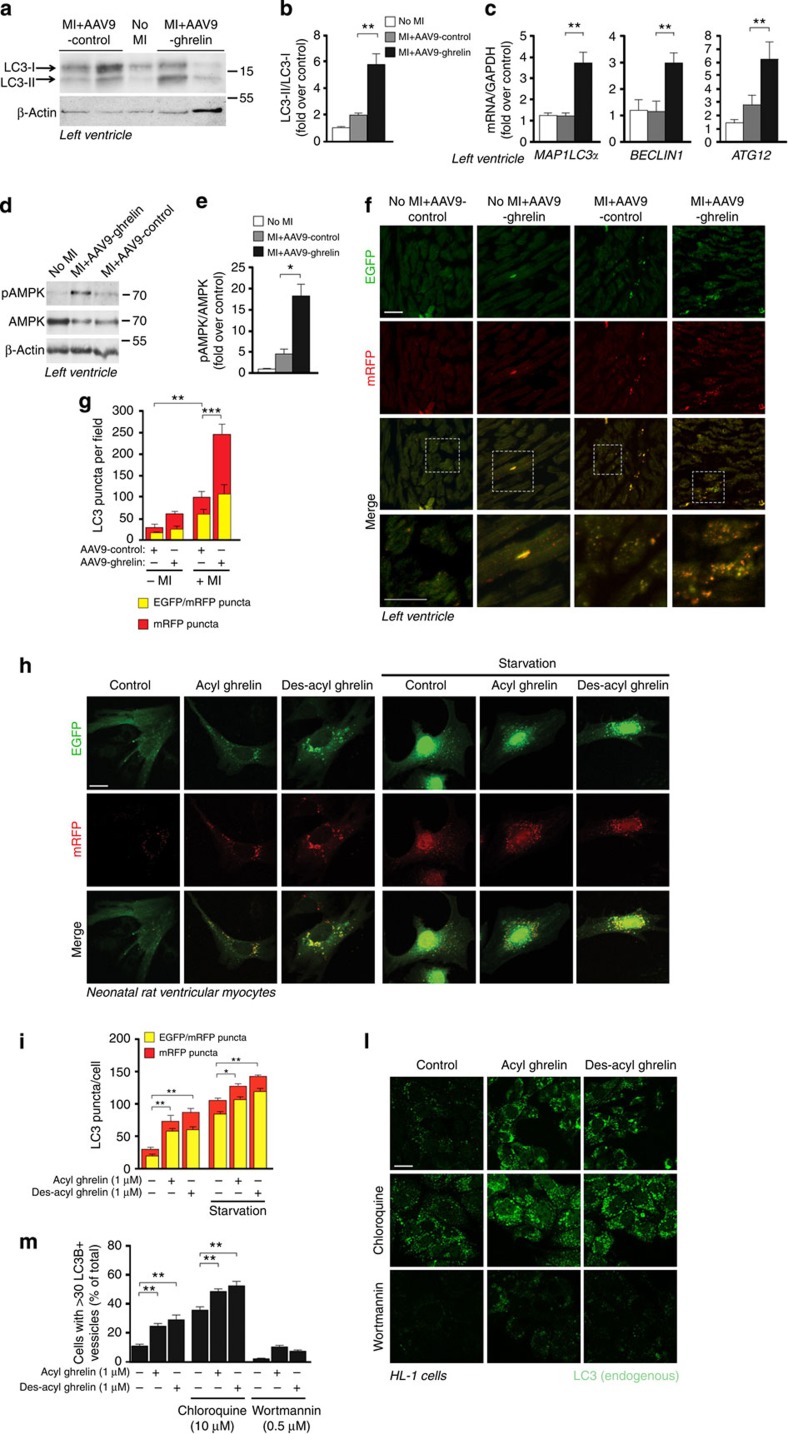
Reduced apoptosis is paralleled by increased autophagy in ischaemic cardiomyocytes overexpressing ghrelin. (**a**,**b**) LC3 lipidation (conversion from LC3-I to LC3-II) in the left ventricles of transduced hearts harvested 2 days after MI. Representative western blot (**a**) and densitometric analysis (**b**; *n*=6). (**c**) Quantification of *MAP1LC3A*, *BECLIN1* and *ATG12* mRNA levels in the left ventricles of transduced hearts at 2 days after MI. Values are normalized for glyceraldehyde 3-phosphate dehydrogenase (GAPDH) and expressed as fold over untreated (*n*=8). (**d**,**e**) AMPK phosphorylation in the left ventricles of transduced hearts harvested 2 days after MI. Representative western blot (**d**) and densitometric analysis (**e**) of total AMPK and phosphor (pAMPK; *n*=6). (**f**) Heart sections of mice injected with AAV9-mRFP-EGFP-LC3 and AAV9-control or AAV9-ghrelin and submitted or not to MI. Autophagosomes appear yellow in the merged image, whereas autolysosomes appear red. Scale bar, 100 μm. (**g**) Quantification of EGFP and mRFP LC3-positive dots per field, using the ImageJ software (*n*=6). (**h**) Primary rat neonatal cardiomyocytes transfected with the mRFP-EGFP tandem fluorescent-tagged LC3 plasmid (ptfLC3) and, 48 h later, treated for 4 h with acyl ghrelin, des-acyl ghrelin (both 1 μM) or vehicle in complete or starving medium. Scale bar, 10 μm. (**i**) Quantification of EGFP and mRFP LC3-positive dots per cell, using the ImageJ software (*n*=30 cells per group). (**l**) Representative immunofluorescence staining for LC3B (green) in HL-1 cells treated for 4 h with acyl ghrelin, des-acyl ghrelin (both 1 μM) or vehicle in the presence or absence of chloroquine (10 μM) or wortmannin (0.5 μM). Scale bar, 50 μm. (**m**) Quantification of the cells with a high number (>30) of LC3B-positive vesicles, expressed as % of the total number of cells (*n*=30 cells per group). All values are mean±s.e.m. One-way analysis of variance (ANOVA) and Bonferroni/Dunn’s *post hoc* test were used to compare multiple groups (**b**,**c**,**e**,**g**,**i**,**m**). *P<0.05, **P<0.01, ***P<0.001 relative to control.

Next, we wondered how ghrelin exerted its protective effect after MI. Multiple evidence indicates that a powerful mechanism protecting the heart against ischaemic damage is macroautophagy (hereafter referred to as autophagy)^13^. In the infarcted hearts transduced with AAV9-ghrelin, we found an increased conversion of the soluble LC3-I protein to lipid-bound LC3-II, which associated with the formation of autophagosomes (representative blots and quantification for AAV9-ghrelin and AAV9-control hearts in Fig. 5a,b, respectively). Consistent with this observation, the mRNAs of genes involved in the autophagic pathway, including *BECLIN1*, *MAP1LC3A* and *ATG12*, were all significantly induced in the AAV9-ghrelin-transduced, infarcted hearts (Fig. 5c). In the same hearts, the AMP-activated protein kinase (AMPK), a key regulator of energy metabolism, which also regulates cardiac autophagy^23^, was also found to be activated (Fig. 5d,e).

To directly visualize the autophagic flux in the infarcted hearts, we generated an AAV9 vector expressing the monomeric red fluorescent protein (mRFP)-enhanced green fluorescent protein (EGFP) tandem fluorescent-tagged LC3 protein, derived from the ptfLC3 plasmid^24^, in which green, but not red, fluorescence is sensitive to the acidic pH within the autolysosome. This vector was administered, together with AAV9-control or AAV9-ghrelin (1 × 10^11^ vg per animal for each vector), immediately after left anterior descending (LAD) coronary artery ligation (*n*=6 per group), or in non-infarcted animals (*n*=6 per group). At day 7 after transduction, the number of LC3-positive vesicles, and, in particular of those showing only red fluorescence, was significantly increased in infarcted hearts injected with AAV9-ghrelin (Fig. 5f,g). This indicated that ghrelin stimulates autophagic flux.

In the heart, the levels of the GOAT enzyme are low^5^ and thus the observed induction of *in vivo* autophagy was probably mediated by unmodified ghrelin. To clarify the relative contribution of the acyl and des-acyl forms of the protein in the promotion of autophagy, we treated rat neonatal cardiomyocytes, previously transfected with the ptfLC3 plasmid, with either of the two peptides. Both of these, but most importantly, des-acyl ghrelin, increased the number of autophagosomes and promoted autophagic flux; this effect was evident both in complete medium and upon serum and glucose starvation, a condition known to increase autophagy (Fig. 5h,i). Superimposable results were obtained using the HL-1 cardiac cell line (Supplementary Fig. 5a,b).

Both acyl and des-acyl ghrelin also increased the levels of endogenous LC3 lipidation in HL-1 cells (9.8±1.3% cells containing more than 30 LC3-positive vesicles in basal conditions versus 23.8±1.7% and 27.3±2.5% after treatment with the two peptides, respectively; Fig. 5l,m). When the cells were treated with the lysosomotropic agent, chloroquine, both peptides still increased vesicle formation (number of LC3B-positive cells: 37.3% in the absence of treatment; 44.4% and 47.2% after addition of acyl or des-acyl ghrelin, respectively), thus confirming that both forms of ghrelin were capable of inducing the autophagic flux. Finally, cells treatment with the phosphatidylinositide 3-kinase (PI3K) inhibitor wortmannin blocked autophagosomes formation in both acyl and des-acyl ghrelin-treated HL-1 cells.

Collectively, these results indicate that ghrelin and, in particular its unmodified form, is an effective inducer of autophagy in isolated cardiomyocytes and *in vivo*, thus suggesting a mechanism of action independent from GHSR-1a receptor activation.

### Ghrelin stimulates autophagy after hindlimb ischaemia

We also studied the effect of ghrelin in inducing autophagy in the skeletal muscle after hindlimb ischaemia. In CD1 mice transduced with AAV9-ghrelin at 7 days after femoral artery resection (cf. above), the amount of p62, a protein rapidly degraded during autophagy, was significantly reduced in comparison to AAV9-control-transduced mice (Supplementary Fig. 6a and b), while the mRNA levels of *BECLIN1*, *MAP1LC3A* and *ATG12* were all induced (Supplementary Fig. 6c). As in cardiac myocytes, both acyl and des-acyl ghrelin induced autophagic flux in C2C12 myoblasts, as concluded by the increased number of mRFP-LC3 autolysosomes in cells transfected with the ptfLC3 plasmid (Supplementary Fig. 6d,e). Finally, in cells treated with chloroquine to induce accumulation of autophagosomes, ghrelin significantly increased conversion of LC3-I to LC3-II (Supplementary Fig. 6f,g).

### Autophagy inhibition blocks the protective effect of ghrelin

**Figure 6:**
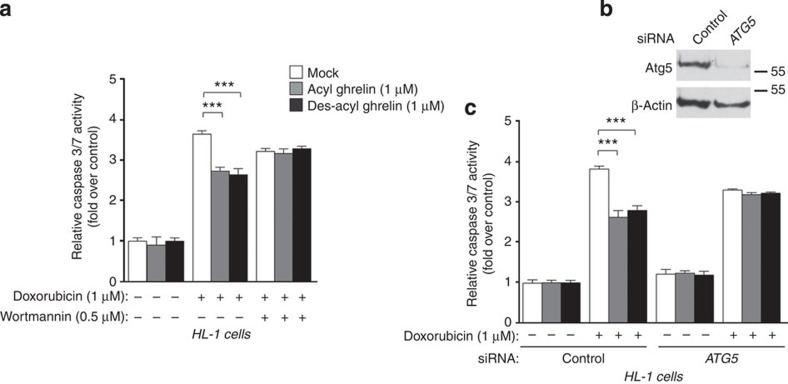
Inhibition of autophagy abolishes the protective effect of ghrelin. (**a**) Pretreatment with wortmannin blocks the protective effect of ghrelin. Activation of caspase 3/7 in HL-1 cells treated with doxorubicin (1 μM) for 20 h in the presence of either acyl or des-acyl ghrelin (1 μM) and with or without wortmannin (0.5 μM). Treatment with wortmannin was initiated 1 h before the others (*n*=8 experiments of 10 replicates each). (**b**,**c**) Atg5 knockdown blocks the protective effect of ghrelin. Representative western blot showing reduced Atg5 protein after siRNA knockdown in HL-1 cells (**b**) and levels of caspase 3/7 activation after RNAi and cell treatment with acyl or des-acyl ghrelin (1 μM) and treatment with doxorubicin (1 μM) for 20 h (**c**; *n*=8 experiments of 10 replicates each). All values are mean±s.e.m. One-way analysis of variance and Bonferroni/Dunn’s *post hoc* test were used to compare multiple groups (**a**,**c**). ***P<0.001 relative to control.

To investigate whether there was a direct correlation between the anti-apoptotic effect of ghrelin an its capacity to induce autophagy, we inhibited autophagy in HL-1 cells using either wortmannin or an small interfering RNA (siRNA) against *ATG5*, essential for the initial phase of formation of autophagic vesicles^25^, and then evaluated the outcome of these treatments on the anti-apoptotic effects of ghrelin. In both conditions, the inhibition of autophagy abrogated the protective effects of both acyl and des-acyl ghrelin against doxorubicin-induced apoptosis (20 h, 1 μM), as assessed by measuring caspase 3/7 activation (Figs 6a for wortmannin and Fig. 6b,c for RNA interference). These results indicate that autophagy is essential in mediating the survival effect of ghrelin on cardiomyocytes.

### Ghrelin removes dysfunctional mitochondria

**Figure 7:**
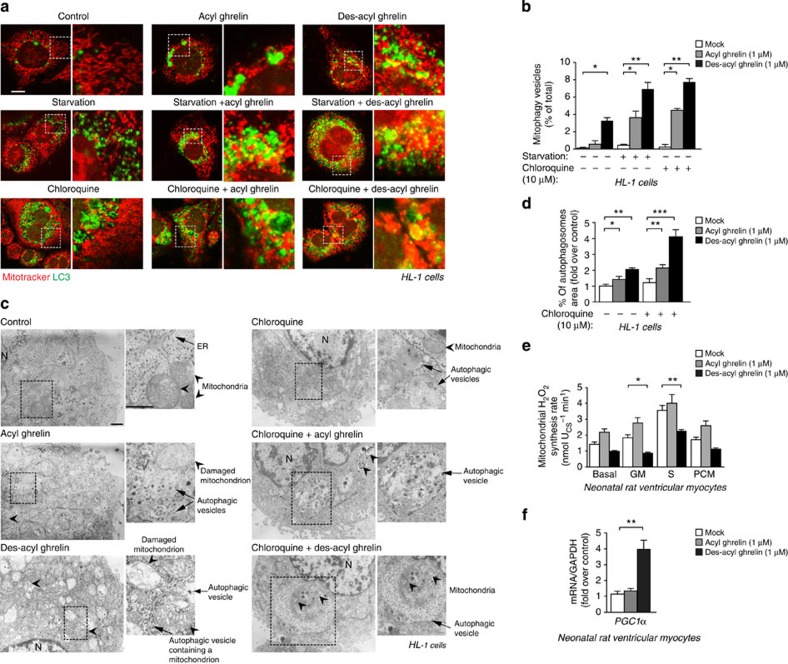
Ghrelin reduces ROS accumulation removing dysfunctional mitochondria. (**a**) Hl-1 cells were incubated in normal or starving conditions with acyl ghrelin, des-acyl ghrelin (1 μM) or vehicle in the presence or absence of chloroquine (10 μM) for 4 h. Mitochondria were labelled with MitoTracker Orange for 30 min at 37 °C. Cells were then fixed, stained for LC3B and imaged using a confocal laser-scanning microscope. Representative images of mitochondria (red), autophagosomes (LC3B, green) and their overlap are shown. For each picture, an enlargement of the overlap image is displayed, showing the presence or absence of co-localizing mitochondria and autophagosomes. Scale bar, 10 μm. (**b**) Quantification of the percentage of LC3B-positive autophagosomes co-localizing with mitochondria (*n*=30 cells per group). (**c**) Representative electron micrographs of HL-1 cells incubated in complete medium with acyl ghrelin, des-acyl ghrelin (both 1 μM) or vehicle in the presence or absence of chloroquine (10 μM) for 4 h. For each picture, an enlargement of the image is displayed, showing the presence of normal or damaged mitochondria (indicated by arrowheads) and autophagic vesicles (indicated by arrows). Scale bar, 1 μm. (**d**) Quantification, from the electron microscopy pictures, of the cytoplasmic areas containing autophagic structures per section. Values are expressed as percentages and normalized over mock conditions (*n*=10 per condition). (**e**) Quantification of reactive oxygen species (ROS) production in intact mitochondria isolated from neonatal rat cardiomyocytes after 48 h of starvation in the absence or presence of acyl or des-acyl ghrelin (both 1 μM). After addition of either ultrapure H_2_O or respiration substrates, state 4-mitochondrial respiration H_2_O_2_ production was measured, *n*=4 (GM, glutamate/malate; S, succinate; PCM, palmitoyl-L-carnitine/malate). (**f**) Quantification of *PGC1a* mRNA expression levels in rat neonatal cardiomyocytes treated with acyl or des-acyl ghrelin (both 1 μM). Values are normalized for glyceraldehyde 3-phosphate dehydrogenase (GAPDH) and expressed as fold over untreated (*n*=4). All values are mean±s.e.m. One-way analysis of variance and Bonferroni/Dunn’s *post hoc* test were used to compare multiple groups (**b**,**d**,**e**,**f**). *P<0.05, **P<0.01, ***P<0.001 relative to control.

One of the specific functional consequences of the induction of autophagy is the removal of dysfunctional cellular organelles. In particular, in the heart, the elimination of swollen and malfunctioning mitochondria by autophagy (mitophagy) is essential to maintain tissue homeostasis^26^. We found that treatment of HL-1 cardiomyocytes with acyl and even more with des-acyl ghrelin markedly increase the number of autophagic vesicles containing mitochondria. This effect was particularly evident after starvation (representative images of LC3-positive vesicles containing autophagocytosed mitochondria and quantifications in Fig. 7a,b, respectively). The peptides still increased the number of mitophagy vesicles in the presence of chloroquine, again indicating stimulation of the mitophagic flux.

We further investigated the induction of autophagy and, in particular, the removal of damaged mitochondria by transmission electron microscopy in HL-1 cells. We found that acyl and, in an even more pronounced manner, des-acyl ghrelin increased autophagy in both complete medium and under starvation (Fig. 7c and Supplementary Fig. 7a, respectively, for representative images and Fig. 7d and Supplementary Fig. 7b for quantification). Of note, clear mitochondrial structures included in autophagic vesicles were detected upon des-acyl ghrelin stimulation in both culture conditions. Upon treatment with chloroquine, numerous mitochondrial structures at different stages of degradation were detected as included in autophagosomes in cells treated with both acyl and des-acyl ghrelin. Chloroquine also favoured the formation of large-size autophagic vesicles containing cellular components; in particular, in cells stimulated with des-acyl ghrelin, damaged mitochondria were identifiable within very large autophagosomes (Fig. 7c and Supplementary Fig. 7a). Taken together, these electron microscopy results clearly confirm the property of the two peptides, and, in particular, of des-acyl ghrelin, to trigger autophagy and the removal of dysfunctional mitochondria.

Dysfunctional mitochondria determine macromolecular damage by increased reactive oxygen species (ROS) production^27^. Consistent with the protective role of ghrelin-induced autophagy of dysfunctional mitochondria, intact mitochondria from starved neonatal cardiac ventricular myocytes treated with des-acyl ghrelin generated significantly less H_2_O_2_ (an indicator of ROS generation) using either glutamate and malate or succinate as oxidative substrates (Fig. 7e). No significant effect on ROS production was exerted by acyl ghrelin in this assay. Moreover, we observed a prominent upregulation of PGC1*α* transcript induced by des-acyl ghrelin, indicating synthesis of novel mitochondrial components, to replace degraded mitochondria (Fig. 7f).

This capacity of ghrelin to remove dysfunctional mitochondria is fully consistent and may well support the observed effect of the peptide in the protection of the infarcted myocardium.

## Discussion

In contrast to phenotypic selection (for example, using phage display libraries), functional screening of genetic libraries *in vivo* is in its infancy. The generation of cDNA^28^,^29^,^30^, ribozyme^31^,^32^,^33^ and short hairpin RNA^34^,^35^ libraries has been achieved in gammaretroviral vectors. However, these vectors only transduce replicating cells, whereas most cells in the body lie outside the cell cycle. Libraries generated in lentiviral vectors can be screened *in vivo*, but their utilization is limited to specific permissive cell types^36^,^37^, which do not include those of interest in the cardiovascular system (cardiomyocytes, skeletal muscle fibres and endothelial cells). First-generation adenoviral vectors have also been engineered to express cDNA libraries^38^,^39^,^40^, but their high immunogenicity *in vivo* leads to the rapid elimination of the transduced cells, thus preventing long-term screenings^41^,^42^,^43^.

Here, for the first time, we report on the feasibility of an *in vivo* functional selection strategy (FunSel), which can be performed over relatively long periods of time and in tissues that are usually refractory to conventional gene transfer. This is rendered possible by the use of AAV vectors, which show exquisite tropism for cardiomyocytes, skeletal muscle fibres, retinal cells, neurons and β-cells in the pancreas (reviewed in ref. 44). Of note, these cell types are the major targets of highly prevalent degenerative disorders, including heart failure, blindness, diabetes and neurodegeneration. Our results indicate that: (i) multiple AAV vectors can be packaged simultaneously, (ii) the infectivity of each vector in such mixtures is maintained *in vivo*, (iii) transgene expression is proportional to vector dilution in the transducing preparation and (iv) vector pool complexity up to 50 vectors per pool allows relatively robust levels of transgene expression. Our choice to screen for secreted factors poses an additional hurdle to the screening process itself, as a putatively positive factor is expected to exert its beneficial effect beyond the transduced cells, a possibility that we also took into account in our mathematical simulations. Our choice, however, was dictated by the eventual possibility of administering the identified factor as a recombinant protein or synthetic peptide, without necessarily relying on viral vector delivery.

FunSel is based on an *in vivo* competitive process between a beneficial gene and those that are neutral or detrimental within the same pool; as the procedure is iterative, the ultimate factor identified is the best performer in the pool. The survival advantage upon which FunSel is based does not necessarily coincide with a direct effect of the transgene on the transduced cells through the regulation of genes directly promoting cell survival. Instead, the effect of the selected transgene might be indirect, for example, ensuing as the consequence of improved blood perfusion, or the regulation of inflammation or immune reactivity, or due to the multiplication of the transduced cells at the expense of other cells, to name but a few of the possible selection mechanisms. *In vivo*, most factors exert their function through pleiotropic mechanism; FunSel thus offers the advantage of screening for beneficial effects that are cumulative. Indeed, here the application of FunSel to search for factors selected after acute ischaemia led to the identification of ghrelin as a protein that induces autophagy, protects from apoptosis and ameliorates energy metabolism, three functions that are strictly connected and eventually improve function under ischaemic conditions.

Converging evidence over the last few years has indicated that autophagy exerts beneficial effects in the heart^17^. Cardiomyocytes have a very long life, often coinciding with the whole life of the organism^45^, and an extremely high metabolic demand. In these cells, the recycling of dysfunctional organelles, in particular mitochondria, is essential to maintain cardiac energy homeostasis and to preserve function and vitality under stress. Loss of autophagy in Atg5^−/−^ knock out mice^14^ and in patients with an inherited defect in the lysosomal protein Lamp-2 (ref. 20) leads to cardiomyopathy and heart failure; the proapoptotic kinase Mst1 induces cardiac dysfunction by impairing autophagy in cardiomyocytes^19^. On the contrary, induction of autophagy protects against ischaemia/reperfusion injury^18^,^46^, limits cell death and tissue damage following acute MI^47^, represents a pro-survival mechanism in chronically ischaemic myocardium^48^ and constitutes a quality control mechanism to remove damaged mitochondria^49^. Thus, the promotion of the autophagic response over time stands as a novel, therapeutic option to mitigate post-infarction LV dilation and cardiac dysfunction. In line with these notions, our observations indicate that hearts expressing ghrelin show highly reduced cardiomyocyte apoptosis immediately after coronary artery ligation and persistence of autophagic flux in the infarct border zone at later times; both findings correlate with markedly improved cardiac function up to 3 months after MI. The short-term, antiapoptotic effect of ghrelin is consistent with previous findings on isolated cardiomyocytes and cardiomyocyte cell lines^50^.

As the original discovery of ghrelin as a peptide with orexigenic properties^6^, it soon became evident that this molecule also exerts beneficial function in the cardiovascular system, including improved inotropic response, cardioprotection and reduction of the inflammatory response (reviewed in ref. 51); patients with heart failure receiving acyl ghrelin (intravenous, twice a day for 3 weeks) showed significantly improved cardiac function^52^. Our results now provide a mechanism by which ghrelin exerts its long-term benefit in the heart.

Our novel finding that ghrelin induces autophagy and, in particular, the removal of dysfunctional mytochondria in cardiomyocytes is consistent with a number of biochemical studies showing that ghrelin activates various signal transduction pathways in these cells (including MAP kinases, PI3K/Akt and AMPK^50^,^53^,^54^,^55^,^56^) also known to regulate at various extents these processes. In particular, we found that infarcted hearts treated with ghrelin showed increased AMPK activity, an observation that is fully consistent with the capacity of this kinase to limit apoptotic activity and stimulate protective cardiac autophagy after ischaemic injury^23^. Previous work has shown that AMPK also triggers the removal of damaged mitochondria and induces mitochondria biogenesis through *PGC1α* activation^57^, an observation in keeping with the increased levels of *PGC1α* we observed in the ghrelin-treated hearts.

Which receptor mediates the autophagic response of skeletal muscle fibres and cardiomyocytes to ghrelin? Mounting evidence over the last few years has indicated that several of ghrelin activities are not mediated by its only discovered receptor, GHSR-1a, which is selective for acyl ghrelin and does not bind the native peptide^6^. Indeed, both acyl and des-acyl ghrelin inhibit apoptosis and activate the PI3K/Akt pathway in H9c2 cardiomyocytes, which do not express GHSR-1a, and protect the skeletal muscle of GHSR-1a knockout mice from fasting-induced atrophy^50^,^55^. In our study, induction of autophagy was observed *in vivo* in the heart, where AAV vectors express the native protein and the GOAT enzyme is poorly expressed. In addition, both autophagy and mitophagy in cultured myocardial cells were stimulated more efficiently by des-acyl than by acyl ghrelin. Taken together, these findings strongly indicate that the pro-autophagic activity of ghrelin is mediated by a yet unidentified receptor, distinct from GHSR-1a. Fully consistent with these observations, the effects of AAV9-ghrelin are maintained after MI in GHSR-1a knockout mice (unpublished observations). Surprisingly, only des-acyl ghrelin protects cardiomyocytes from ROS induced by energetic substrates. This finding is in keeping with previous data reporting that des-acyl ghrelin, but not acyl ghrelin, prevents ischaemia-induced ROS production in skeletal myocytes and upregulates anti-oxidant enzymes^58^.

The identification of the exact molecular pathways triggering myocardial autophagy by des-acyl ghrelin will await the identification of the long sought, but still elusive, receptor binding of this native form of the peptide.

## Methods

### Generation of the AAV library

All cDNAs were amplified from the original cloning plasmids (modified pBluescript SK (+)/modified Bluescript1 and pFlcI) by PCR with specific primers (reported in Supplementary Table 3), to insert the *Xho*I and *Not*I restriction sites at the 5′ and 3′ ends of each cDNA. The amplified sequences were individually cloned into the pZac2.1 vector (Gene Therapy Program, Penn Vector core, University of Pennsylvania, USA) under the control of the CMV IE promoter. The AAV-control vector carried an empty polylinker. The nucleotide sequence of each clone was verified.

### Production and purification of AAV vectors

The AAV vectors were prepared by the AAV Vector Unit at the ICGEB Trieste (http://www.icgeb.org/avu-core-facility), according to established procedures^59^. Briefly, AAV vectors of serotypes 2 and 9 were generated in HEK293T cells, using a triple-plasmid co-transfection for packaging. Viral stocks were obtained by CsCl_2_-gradient centrifugation. Titration of AAV viral particles was performed by real-time PCR quantification of the number of viral genomes, measured as CMV copy number. The viral preparations had titre between 1 × 10^13^ and 5 × 10^13^ vg per ml.

### Animal studies

Animal care and treatment were conducted in conformity with institutional guidelines in compliance with national and international laws and policies (EEC Council Directive 86/609, OJL 358, 12 December 1987), upon approval by the ICGEB Animal Welfare Board and Ethical Committee as well as by the Italian Minister of Health. CD1 mice and Wistar rats were obtained from Harlan and maintained under controlled environmental conditions.

### *In vivo* AAV library screening

Pools of plasmids, mixed in equimolar amounts, were used for batched AAV2 vector production. Each vector preparation (30 μl, containing 1 × 10^10^ vg per leg) was injected bilaterally into the tibialis anterior muscle of 3-month-old female CD1 mice (*n*=12); 2 weeks later the animals were submitted to unilateral femoral artery resection as detailed in the next paragraph. After 2 additional weeks, the animals were killed and DNA was extracted from the injected muscles of both legs using the DNeasy Blood and Tissue kit (Qiagen). The cDNA inserts from the extracted DNA were amplified by 25 PCR cycles using primers annealing on the common pZac2.1 vector sequence and flanking the multiple cloning site. Primer sequences (pZac2.1 FW and RV) are reported in Supplementary Table 3. Part of the amplified DNA was visualized by 1% agarose gel electrophoresis (Fig. 1c); the rest was digested with *Xho*I and *Not*I and re-cloned into the pZac2.1 vector to generate a new AAV9 vector preparation. The cycle was repeated for a total of three times.

### Hindlimb ischaemia

For *in vivo* AAV vector selection, the AAV9-Pool vector (30 μl, 1 × 10^10^ vg per animal) was bilaterally delivered to the tibialis anterior muscles of 3-month-old female CD1 mice (*n*=12). After 15 days, animals were submitted to unilateral femoral artery resection.

For the analysis of AAV9-ghrelin effects, the same procedure was adopted, but 50 μl of recombinant AAV vectors (AAV9-control or AAV9-ghrelin; 1 × 10^11^ vg per animal) were injected (female 3-month-old CD1 mice, *n*=25 per group).

Before femoral artery resection, CD1 mice were anesthetized by i.p. injection of ketamine (100 mg kg^−1^) and xylazine (10 mg kg^−1^) and placed on a warming pad to maintain core body temperature at 37 °C. Unilateral hindlimb ischaemia was induced by resecting a 2.5-cm segment of the left femoral artery. The tibialis anterior muscles of animals injected with AAV9-control or AAV9-ghrelin were collected at 7 (*n*=7 per group) and 21 days (*n*=18 per group) after resection. *In vivo* evaluation of limb function and ischaemic damage was performed at days 2, 7 and 21 post ischaemia by assigning a numeric score, according to established criteria^60^.

### Myocardial infarction

MI was produced in female CD1 mice (8to 10-week old), by permanent LAD coronary artery ligation as already described^61^. In details, mice were anesthetized with ketamine (100 mg kg^1^) and xylazine (10 mg kg^−1^), placed on a warming pad to maintain body temperature at 37 °C and endotracheally intubated and connected to a rodent ventilator (Model 131, Nemi Scientific Inc.). The beating heart was accessed via a left thoracotomy; after removing the pericardium, the heart was exposed and the descendent branch of the LAD coronary artery was visualized by a stereomicroscope (Leica) and ligated with an 8-0 silk suture. Ligation was confirmed by the whitening of the anterior wall of the LV. Immediately after LAD ligation, 30 μl of recombinant AAV vector (AAV9-ghrelin or AAV9-control; 1 × 10^11^ vg per animal; *n*=25 per group) were administered into the infarct border zone using a tuberculin syringe (30 G; Roche). The chest was closed and the mice moved to a prone position on a heating pad until they recovered spontaneous breathing. Echocardiography, as detailed below, was performed after 2, 7, 30, 60 and 90 days and the hearts were collected at 2 (*n*=8 animals per group), 7 (*n*=5 animals per group) and 90 days (*n*=12 animals per group) after infarction.

### Echocardiography

To analyse LV function and dimension, transthoracic two-dimensional echocardiography was performed in mice sedated with 5% isoflurane, using the Vevo 770 Ultrasound (Visualsonics), equipped with a 30-MHz linear array transducer. M-mode tracing were used to measure LV anterior and posterior wall thickness and LV internal diameter at end-systole and end-diastole in short axis view, which were used to calculate fractional shortening and ejection fraction.

### Histology and immunofluorescence

At the end of the studies, animals were killed and muscles or hearts were collected and washed in PBS. Tissue samples were snap frozen or fixed in 4% formaldehyde and embedded in paraffin for immunofluorescence or histological analysis. Frozen sections of the tissues were used for TUNEL assay (described below), whereas paraffin-embedded sections of muscles recovered at day 21 post ischaemia or hearts recovered at day 7 post MI, were stained with haematoxylin-eosin for inflammatory cell infiltration analysis or evaluation of ischaemic lesion area and fibre regeneration after hindlimb ischaemia (four randomly chosen fields per tissue transversal section were quantified using the ImageJ software). A total of seven animals per group were analysed. The hearts collected 90 days after infarction were submitted to Azan’s trichrome staining (Bio-Optica), according to the manufacturer’s protocol. Infarct size was measured on stained sections as the percentage of (endocardial+epicardial circumference of infarct area) × (endocardial+epicardial circumference of LV) ^−1^. A total of 12 animals per group were analysed.

Vasculature staining was performed on tibialis anterior frozen sections, blocked for 1 h with 2% BSA in PBS; endothelial cells were detected using FITC-conjugated lectin from Triticum vulgaris (L4895 1:200; Sigma) and vascular smooth muscle cells using a Cy3-conjugated anti-*α*-SMA mouse monoclonal antibody (clone 1A4 1:200; Sigma). Nuclei were stained with DAPI (H-1,200, Vector laboratories).

### Western blotting

To quantify the levels of LC3B and pAMPK/AMPK proteins *in vivo*, mice hearts transduced with AAV9-control or AAV9-ghrelin were harvested 2 days after MI, snap frozen in liquid nitrogen and stored at −80 °C before lysis in RIPA buffer (20 mM Tris-HCl, pH 7.4; 150 mM NaCl; 1 mM EDTA; 1 mM EGTA; 0.5% NaDOC; 0.5% NP-40 and 0.1% SDS) containing Protease Inhibitor Cocktail Tablets (Roche), Phosphatase Inhibitor Cocktail (Thermo Scientific) and 1 mM phenylmethyl sulphonyl fluoride. Protein concentration was determined by the Bradford method (Bio-Rad Laboratories). For LC3B detection, total protein extract (50 μg) was resolved on 14% SDS–polyacrylamide gel electrophoresis (SDS–PAGE) and transferred to polyvinylidene difluoride membranes (GE Healthcare). Total protein extract was resolved on 8% SDS–PAGE and transferred to nitrocellulose membranes (GE Healthcare) for pAMPK/AMPK. Immunoblots were blocked in 5% bovine serum albumin in TBS-Tween (50 mM Tris-HCl, pH 7.4; 200 mM NaCl and 0.1% Tween-20), overnight at 4 °C and incubated for 2 h at room temperature with the primary antibodies for LC3B (L7543, 1:2,000; Sigma-Aldrich), pAMPK (2531, 1:1,000; Cell Signalling) or AMPK (2532, 1:1,000; Cell Signalling) and then for 1 h at room temperature with the HPR-conjugated goat anti-rabbit secondary antibody (31,460, 1:5,000; Thermo Scientific). As a gel loading control, the membranes were incubated for 30 min with conjugated mouse monoclonal anti-β-Actin (A3854 1:25,000; Sigma-Aldrich). Proteins were detected by enhanced chemiluminescence (GE Healthcare).

The same lysis procedure was used to obtain total protein extract from tibialis anterior muscles of CD1 mice 7 days after peripheral ischaemia and autophagy induction was evaluated using anti-p62 antibody (GP62-C 1:2,000; PROGEN Biotechnik GmbH) for 2 h at room temperature and then the HPR-conjugated goat anti-guinea pig secondary antibodies (106-035-003 1:5,000; Jackson ImmunoResearch) for 1 h at room temperature. The level of Atg5 protein in HL-1 cells after Atg5 genomic silencing was quantified using the rabbit monoclonal anti-Atg5 antibody (12,994 1:2,000; Cell Signalling). Full-length uncropped images of gels are shown in Supplementary Fig. 8.

### Enzyme immunoassay analysis (EIA)

Intracardiac injection of AAV vectors (AAV9-control or AAV9-ghrelin, *n*=4 per group; 1 × 10^11^ vg per animal) in 8- to 10-week-old female CD1 mice, was performed as described above for animals that underwent MI. Plasma and heart tissues of the injected animals were collected 90 days after AAV injection, tissues were prepared as previously described^62^, and the levels of acyl and des-acyl ghrelin peptides were assayed by specific EIA kits from SPIBio (Bertin Pharma, France), according to the protocol provided by the supplier.

### TUNEL and caspase 3/7 assays for apoptosis detection

TUNEL assay was performed on frozen sections of tibialis anterior muscles collected 7 days after hindlimb ischaemia, on LV infarcted hearts, collected 2 days after MI and on primary neonatal rat cardiomyocytes. Both cells and tissue sections were fixed with 4% paraformaldehyde (PFA) and permeabilized with 0.5% Triton X-100 for 15 min. After 1 h in blocking solution (2% BSA) at 37 °C, heart tissues and primary neonatal rat cardiomyocytes were stained with the primary antibody against sarcomeric α-actinin (EA-53 1:200, Abcam) for 2 h. Alexa Fluor-488 donkey anti-mouse (A-21202 1:500; Life Technology) was used as a secondary antibody. Skeletal muscles were stained with the FITC-conjugated lectin from Triticum vulgaris (L4895, 1:200; Sigma). Apoptotic cells were visualized by *in situ* cell death detection kit, TMR red (Roche Diagnostics), according to the manufacturer’s instructions. Nuclei were stained with DAPI (H-1200, Vector laboratories, CA). All images were acquired by a DMLB upright fluorescence microscope (Leica Mycrosystems) equipped with charge-coupled device camera (CoolSNAP CF, Roper Scientific) using MetaView 4.6 quantitative analysis software (MDS Analytical Technologies). At least five high-magnification fields were counted for each experimental condition.

Apoptosis for rat neonatal cardiomyocytes and HL-1 cells treated with doxorubicin (0.5 and 1 μM, respectively) was evaluated 20 h after treatment using the Caspase-Glo 3/7 Assay System (Promega) according to the protocol provided by the supplier.

### RNA and DNA extraction and quantification

Total RNA from tissue samples was extracted using TRIzol reagent (Invitrogen) according to the manufacturer’s instructions, digested by *DNA*seI and reverse-transcribed using hexameric random primers. Quantification of gene expression was performed by quantitative real-time PCR using the primers and probes reported in the Supplementary Table 3. The murine housekeeping gene *GAPDH* was used to normalize the results. All the amplifications were performed using a BIORAD CFX96 real-time machine, using pre-developed and custom-designed assays (Applied Biosystems) or SYBRGreen (Bio-Rad Laboratories). Quantification of individual vector DNA in muscle samples was performed by Q-PCR using SYBRGreen. A Taqman probe and primers that specifically recognize the CMV promoter, which is common to all the AAV vectors in the study, was used to quantify total viral genomes. Primers and probes for viral DNA quantifications are reported in Supplementary Table 3.

### Neonatal rat cardiomyocyte isolation and treatment

Ventricular myocytes from 1- to 2-day-old Wistar rats were prepared according to a previously described protocol, yielding 90% purity^63^. In details, the hearts were retrieved, the ventricles isolated from atria and vessels and minced in small pieces down to a diameter of ∼2–3 mm. The fragments were dissociated in CBFHH (calcium and bicarbonate-free Hanks with HEPES) buffer containing 2 mg ml^−1^ of trypsin and 20 μg ml^−1^ of DNase II, repeating the digestion in fresh buffer approximately ten times for 10 min each with slow stirring, followed by ten times up and down gentle pipetting of tissue pieces. After each cycle, the supernatant was collected into a solution containing calf serum (10%, v/v) to neutralize the action of trypsin. At the end of the cycles of digestion, the collected cells were centrifuged for 10 min at 1,200 r.p.m. and the cellular pellet was resuspended in DMEM containing 5% fetal bovine serum (FBS). Cells were preplated for 2 h into 100-mm dishes, in order to allow the engraftment on the plate of fibroblasts. By this procedure, stromal cells were separated from unattached myocytes that were then plated in dishes for primary cells. Neonatal rat ventricular myocytes were plated at low density in DMEM high glucose containing 5% FBS, vitamin B12 (2 mg ml^−1^) and penicillin–streptomycin (100 U ml^−1^) and cultured as described previously^64^.

Cardiomyocytes were infected with AAV vectors at a MOI: 5 × 10^4^ vg per cell the day after plating; 12 h later, the culture medium was changed. After additional 12 h, cells were treated with isoproterenol hydrochloride 10 μM (Sigma) for 48 h or doxorubicin hydrochloride 0.5 μM (Sigma) for 24 h. Viability was assessed using the *in situ* cell death detection kit, TMR red (Roche) as detailed in the Supplementary Information. After staining, cells were fixed in 4% PFA and fluorescence was analysed by the ImageXpressMICROTM microscope and the MetaXpress software, both from Molecular Devices.

### Analysis of autophagy in cultured cells

HL-1 atrial cardiomyocyte cells^65^ were kindly provided by Dr William Claycomb (LSU Health Sciences Center, Louisuana) and were cultured on gelatin/fibronectin-coated plates in Claycomb medium (Sigma Aldrich) supplemented with 10% FBS, penicillin–streptomycin (100 U ml^−1^), norepinephrine 0.1 mM and L-glutamine 2 mM. For endogenous autophagy analysis, cells were seeded in four-chamber slides (6 × 10^4^ cells per well) and, after 24 h, treated with murine acyl or des-acyl ghrelin 1 μM (Bachem), for 4 h. To analyse autophagic flux, cells were treated with the peptides in the presence of the lysosomotropic alkalinizing agent chloroquine (Sigma-Aldrich) at a concentration of 10 μM, to inhibit autophagosome-lysosome fusion^66^. As autophagy inhibitor wortmannin (Sigma-Aldrich) 0.5 μM was used. After 4 h, cells were fixed with 4% PFA at room temperature and permeabilized with 0.1% Triton X-100 for 5 min. After 1 h in blocking solution (5% BSA), cells were incubated with the primary rabbit anti-LC3B antibody (L7543, 1:200; Sigma-Aldrich) for 2 h. Alexa Fluor 488 donkey anti-mouse (A-21202, 1:500; Life Technology) was used as secondary antibody. Cells were imaged at × 100 magnification using a Zeiss LSM-510 confocal laser-scanning microscope. The percentage of cells showing numerous LC3 puncta (>30 dots per cell)^67^ was quantified using the cell counter tool of the ImageJ software (NIH). A minimum of 30 cells were scored for each condition.

C2C12 myogenic cells (ATCC CRL-1772) were cultured in high-glucose DMEM plus 10% FBS and penicillin–streptomycin (100 U ml^−1^) and treated for 4 h with murine acyl or des-acyl ghrelin (1 μM) in the presence or absence of chloroquine (10 μM) to evaluate LC3I/II conversion by western blot.

To analyse autophagic flux *in vitro* neonatal rat cardiomyocytes, HL-1 and C2C12 cells were plated in four-chamber slides (1 × 10^5^, 6 × 10^4^ and 2 × 10^4^ cells per well, respectively) and transfected with ptfLC3 using Lipofectamine 2,000 (Life Technologies) according to the manufacturer’s instructions. PtfLC3 was a gift from Tamotsu Yoshimori (Addgene plasmid # 21074). Twenty-four hours after transfection, culture medium was replaced by fresh medium and after additional 24 h cells were treated with acyl or des-acyl ghrelin, either in complete or in starvation medium (DMEM without glucose, sodium pyruvate and FBS). Four hours later, cells were fixed with 4% PFA and imaged at × 100 magnification using a Zeiss LSM-510 confocal laser-scanning microscope. LC3 green and red dots were quantified using the cell counter tool of the ImageJ software. A minimum of 30 cells were scored for each condition.

SiRNA knockdown of Atg5 was performed on HL-1 cells seeded in Primaria 96-well plates (BD Falcon) and transfected at a final concentration of 50 nM with mouse Atg5 siRNA (M-064838-02-0005; Dharmacon) by a standard reverse transfection^61^ using Lipofectamine RNAiMAX (Life Technologies). Twenty-four hours after transfection, culture medium was replaced by fresh medium and after additional 24 h cells were treated with murine acyl or des-acyl ghrelin (1 μM) and doxorubicin (1 μM). Twenty hours later apoptosis, activation was measured using the Caspase-Glo 3/7 Assay System (Promega).

### Autophagic flux analysis *in vivo*

To assess induction of autophagy *in vivo*, we generated an AAV9 vector expressing the mRFP-EGFP tandem fluorescent-tagged LC3, derived from the ptfLC3 plasmid^24^, under the control of CMV promoter. After MI, this vector was administered together with AAV9-control or AAV9-ghrelin (final titer of 1 × 10^11^ vg per animal) into the infarct border zone of 3-month-old female CD1 mice (*n*=6). Animals were killed 7 days later and EGFP and mRFP signals were analysed on frozen sections by confocal microscopy. The tissues were imaged at × 63 magnification using a Zeiss LSM-510 confocal laser-scanning microscope; LC3 green and red dots were quantified using the ImageJ software, as described in the previous paragraph.

### Fluorescence microscopy for mitochondria

To evaluate the formation of autophagic vesicles including mitochondria, HL-1 cells (6 × 10^4^ cells per well) were cultured in four-chamber slides in complete Claycomb medium. After 24 h, cells were starved as described above, treated with 1 μM acyl or des-acyl ghrelin for 4 h with or without chloroquine and then loaded with 500 nM MitoTracker Orange CMTMRos (Life Technologies) at 37 °C for 30 min. Cells were washed with PBS, fixed with 4% PFA and then incubated with the anti-LC3B antibody (L7543, 1:200; Sigma-Aldrich) for 2 h. Alexa Fluor 488 anti-rabbit was used as secondary antibody. Cells were imaged at × 100 magnification using a confocal laser-scanning microscope.

### Transmission electron microscopy

HL-1 cells pellets were fixed in 2% glutaraldehyde (Electron Microscopy Sciences) containing 5 mM CaCl_2_ in 0.1 M cacodylate buffer, pH 7.3, for 1 h at room temperature, rinsed twice for 10 min with the buffer and fixed with 1% osmium tetroxide and 0.8% potassium ferricyanide in the same buffer for 1 h at 4 °C. Pellets were dehydrated in ascending alcohols, treated with propylene oxide and embedded in Araldite (Electron Microscopy Sciences). Ultrathin sections of the samples were cut on a Top Ultra 150 ultramicrotome (Pabish) and collected on 300-mesh copper grids. The grids were stained with uranyl acetate and lead citrate and examined at 80 kV using a Jeol 100S transmission electron microscopy.

### Mitochondrial H_2_O_2_ production

ROS production in intact isolated mitochondria was assessed by quantification of H_2_O_2_ synthesis using the Amplex Red-horseradish peroxidase method^68^ as previously described^69^. Neonatal rat cardiomyocytes were plated in 100 mm^2^ plates (3 × 10^6^ cells); after 2 days, they were starved and treated with acyl or des-acyl ghrelin (1 μM) for 48 h. Cells were then collected and processed by homogenization in Sucrose-Hepes-EGTA (SHE) buffer (260 mmol l^−1^ sucrose, 1 mmol l^−1^ EGTA, 10.5 mmol l^−1^ HEPES, pH=7.2) in ice. Mitochondria were then isolated by differential centrifugation: nuclei and myofibrillar fraction were removed (720*g*, 4 °C, 5 min), and mitochondria centrifuged (10,000*g*, 4 °C, 5 min), washed in buffer SHE and resuspended in Respiration buffer (4 mmol l^−1^ KH_2_PO_4_, 1 mmol l^−1^ MgCl_2_, 14 mmol l^−1^ NaCl, 125 mmol l^−1^ KCl, 20 μmol l^−1^ EGTA, 20 mmol l^−1^ HEPES, pH=7.4). Mitochondrial suspension was added to a 96-well microplate containing in each well 10 volumes assay buffer 10 μmol l^−1^ Amplex Red (Invitrogen), 0.4 U ml^−1^ horseradish peroxidase, 0.2% BSA in buffer R and 1 volume of ultrapure H_2_O, diluted respiration uncoupler (CCCP) or inhibitor (AA). After an incubation of 20 min at 37 °C and the addition of either ultrapure H_2_O or respiration substrate, state 4 mitochondrial respiration H_2_O_2_ production was measured monitoring the conversion of Amplex Red to resorufin using a microplate fluorimeter (Infinite F200, Tecan Group). Working dilutions of respiratory inhibitors and substrates were prepared immediately before use. The final concentrations in the assay were: 8 mmol l^−1^ glutamate, 4 mmol l^−1^ malate; 10 mmol l^−1^ succinate (S); 50 μmol l^−1^ palmitoyl-L-carnitine, 2 mmol l^−1^ malate. The rate of H_2_O_2_ synthesis was calculated by interpolation of resorufin fluorescence variation over time with a calibration curve from H_2_O_2_ standards. Data were normalized for sample mitochondrial content as measured by classic citrate synthase activity assay and expressed as nmol UCS^−1^ min^−1^.

### Statistical analysis

All data are presented as mean±standard error of the mean (s.e.m.). One-way analysis of variance and Bonferroni *post-hoc* test were used to compare multiple groups. In the case of experiments entailing the follow-up of the same animals over multiple time points, we used analysis of variance on repeated measures to analyse statistical significance of the differences between groups over time. Pairwise comparison between groups was performed using the Student’s *t*-test. The Kolmogorov–Smirnov test was used to compare continuous distributions. *P*<0.05 or lower was considered statistically significant. Statistical analysis was carried out using Prism Software (GraphPad).

## Additional information

**How to cite this article**: Ruozi, G. *et al*. AAV-mediated *in vivo* functional selection of tissue-protective factors against ischaemia. *Nat. Commun.* 6:7388 doi: 10.1038/ncomms8388 (2015).

## Supplementary Material

Supplementary Information Supplementary Figures 1-8 and Supplementary Tables 1-3
