# Supplementary material for: AAV-mediated in vivo functional selection of tissue-protective factors against ischaemia
Source: Nat Commun. 2015 Jun 11;6:7388. doi: 10.1038/ncomms8388 (PMC4477044; doi:10.1038/ncomms8388)
Supplement: Supplementary Information — Supplementary Figures 1-8 and Supplementary Tables 1-3 [file ncomms8388-s1.pdf]

SUPPLEMENTARY INFORMATION

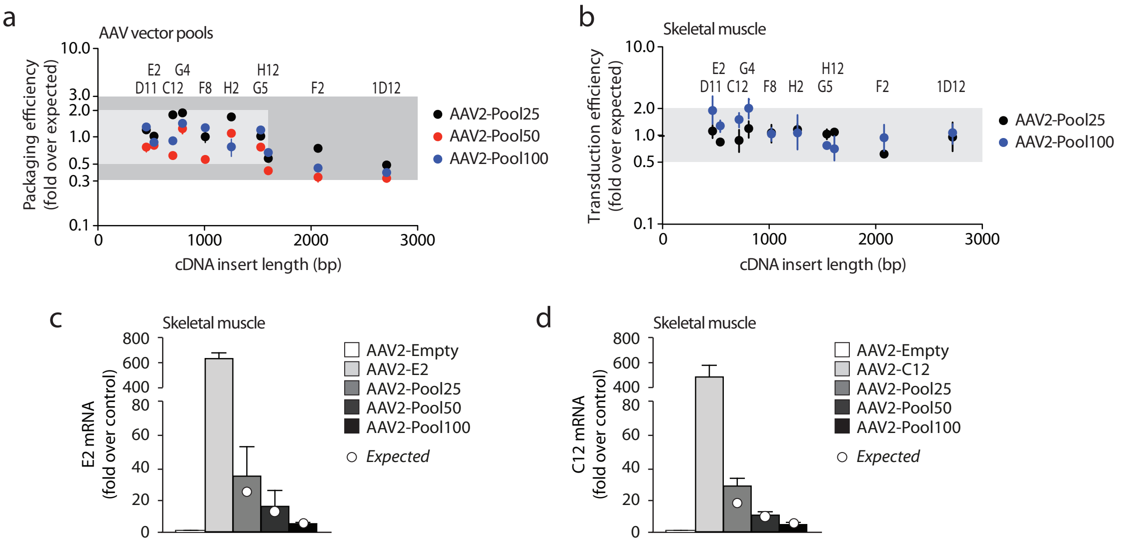

Supplementary Figure 1. Virologic feasibility of FunSel. (**a**) Quantification of 10 selected clones in AAV2 preparations obtained by transfecting packaging cells with equimolar amounts of 25, 50 or 100 vector plasmids, plotted against the length of inserts. Vector codes correspond to the clones listed in **Supplementary Table 2**. Quantification of the input plasmid DNA, before packaging, and of packaged DNA obtained from viral lysates was obtained by real-time PCR using insert-specific primer pairs. The 10 analyzed genes were representative of all clones in the library (range of cDNA inserts: 459-2941 bp). For each clone, results are shown as a ratio between frequency of packaged vector DNA and frequency of plasmid DNA in each pool (theoretical value for each clone: 1). The dark gray area includes values in the ±3-fold relative packaging efficiency range; all analyzed clones fell within this range. The light gray area includes values in the ±2-fold; all clones with cDNAs shorter than 1,500 bp were within this range. Constructs containing cDNA inserts >1500 bp appear disfavoured in competitive packaging. (**b**) Quantification of 10 clones in AAV2-Pool25 and in AAV2-Pool100 after muscle transduction. The two Pools ($5x10^{10}$ vg) were injected into the tibialis anterior muscle of CD1 mice (n=4); three days after transduction, total DNA was extracted and the copy number of the same 10 clones as in panel **a** determined, after standardization for total AAV DNA. For each clone, results are shown as a ratio between frequency of vector in DNA from muscle and frequency of packaged DNA.All analyzed clones fell within the boxed area which includes values in the ±2-fold relative transduction range. (**c,d**) Quantification of mRNA levels of the E2 (**c**) and C12 (**d**) transgenes in mouse tibialis anterior muscle transduced with a control AAV2-Empty vector, AAV2-Pool25, AAV2-Pool50, AAV2-Pool100 and either AAV2-E2 or AAV2-C12 (n=3 per group). All animals received $1x10^{11}$ vg; the expression levels of the two transgenes were analyzed 15 days after transduction and normalized for GAPDH mRNA. In both cases, there was no statistical difference between observed and expected values (white dot), as determined according to vector dilution. All values are mean±SEM.

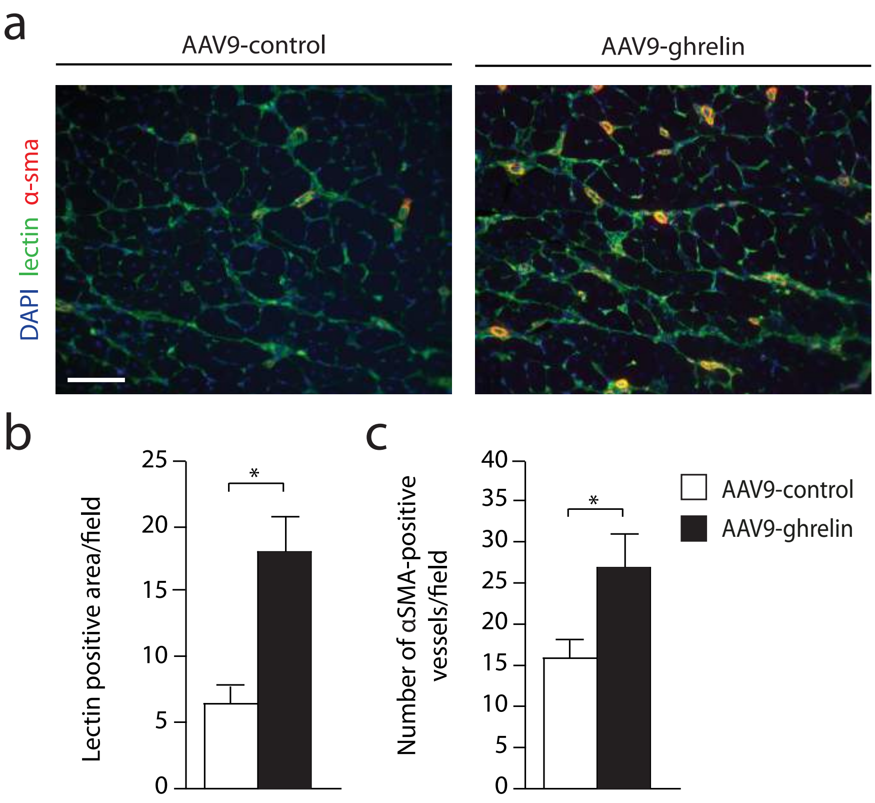

Supplementary Figure 2. AAV9-ghrelin preserves vasculature structures in ischemic muscle. (**a**) Visualization of vessels by immunofluorescence of ischemic tibialis anterior muscle at day 21 after femoral artery resection in AAV9-control and AAV9-ghrelin injected animals (green, lectin positive-endothelial cells; red, α-SMA cells; blue, cell nuclei). Scale bar=100 µm. (**b, c**) Quantification of capillary density as FITC-lectin positive vessels per mm^2 (**b**) and α-SMA positive vessels per field (**c**) in AAV9-control and AAV9-ghrelin injected muscles (n=5). All values are mean±SEM. Pairwise comparison was performed with the Student's t test *P<0.05.

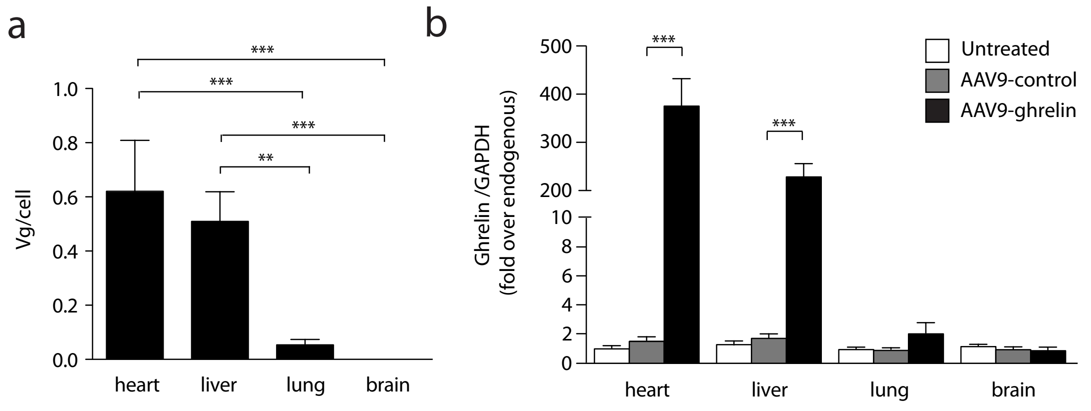

Supplementary Figure 3. Biodistribution of vector genomes and transgene expression levels 15 days after intracardiac injection of AAV9-ghrelin or AAV9-control in adult CD1 mice. (**a, b**) Real Time PCR quantification of viral genomes (vg) DNA per cell (**a**) and quantification of ghrelin mRNA (**b**) in heart, liver, lung and brain of injected mice (n=4 per group). Expression values are normalized for GAPDH and expressed as fold over endogenous. One-way ANOVA and Bonferroni/Dunn's post hoc test were used to compare multiple groups **P<0.01, ***P<0.001 relative to control.

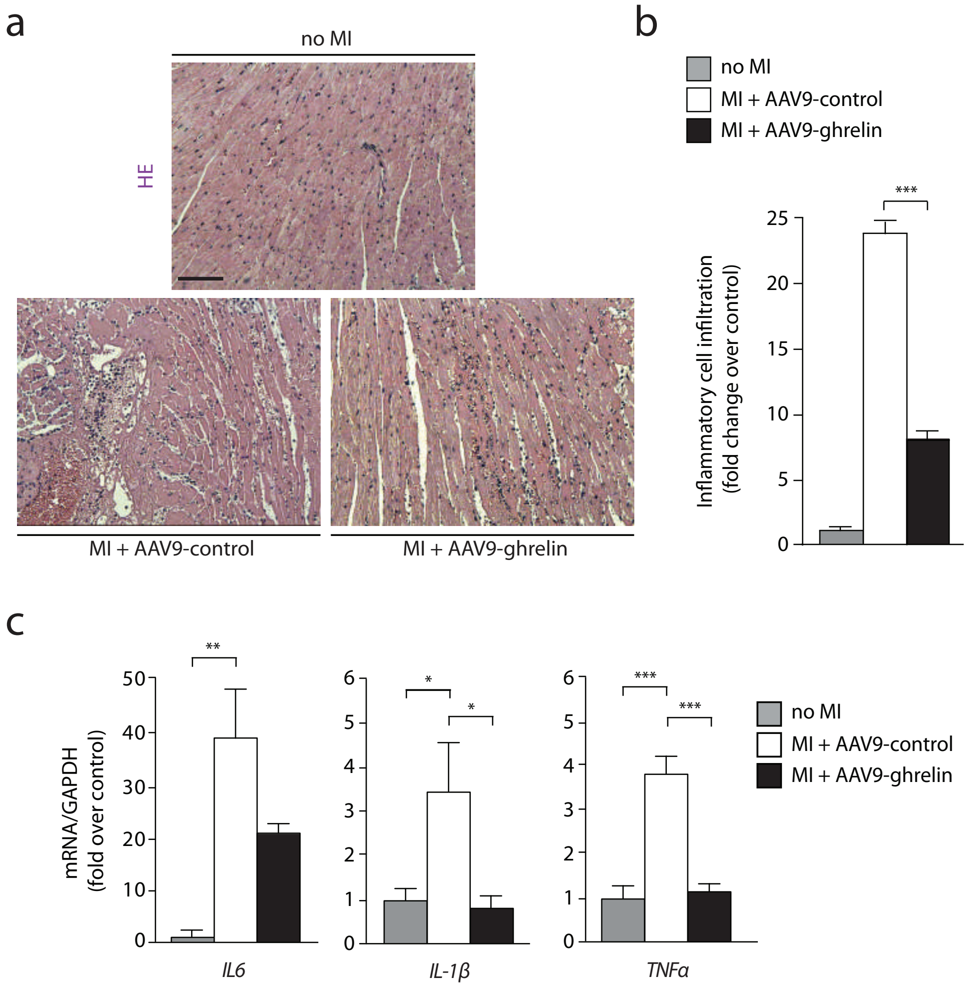

Supplementary Figure 4. AAV9-ghrelin reduces inflammation after myocardial infarction. (**a**) Hematoxylin and eosin staining of heart sections two days after MI and intracardiac delivery of either AAV9-ghrelin or AAV9-control. Scale bar=100 µm. (**b**) Quantification of inflammatory cell infiltration, calculated as relative increase over normal conditions (without MI), n=5. (**c**) Expression levels of inflammatory cytokines, analyzed by real-time PCR, at day at day 2 after MI. Values are normalized for GAPDH and expressed as fold over untreated (n=5). All values are mean±SEM. One-way ANOVA and Bonferroni/Dunn's post hoc test were used to compare multiple groups *P<0.05, **P<0.01, ***P<0.001 relative to control.

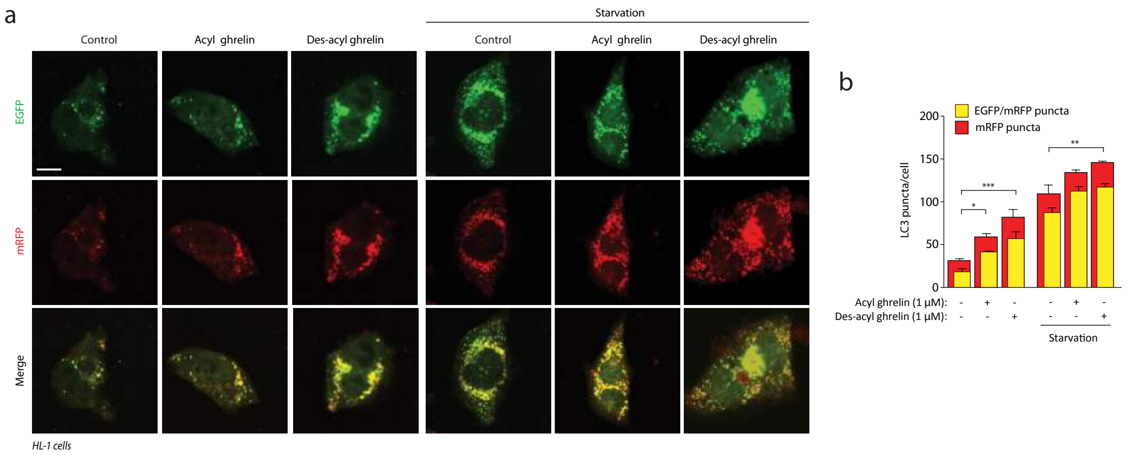

Supplementary Figure 5. Ghrelin induces autophagy in HL-1 cells. (**a**) HL-1 cells transfected with the mRFP-EGFP tandem fluorescent-tagged LC3 plasmid (ptfLC3) and, 48 hours later, treated for 4 hours with acyl ghrelin, des-acyl ghrelin (both 1 µM) or vehicle in complete or starving medium. Scale bar=10 µm. (**b**) Quantification of EGFP and mRFP LC3 positive dots per cell, using the ImageJ software (number of analyzed cells: 30 per group). All values are mean±SEM. One-way ANOVA and Bonferroni/Dunn's post hoc test were used to compare multiple groups $^{*}P<0.05$, $^{**}P<0.01$, $^{***}P<0.001$ relative to control.

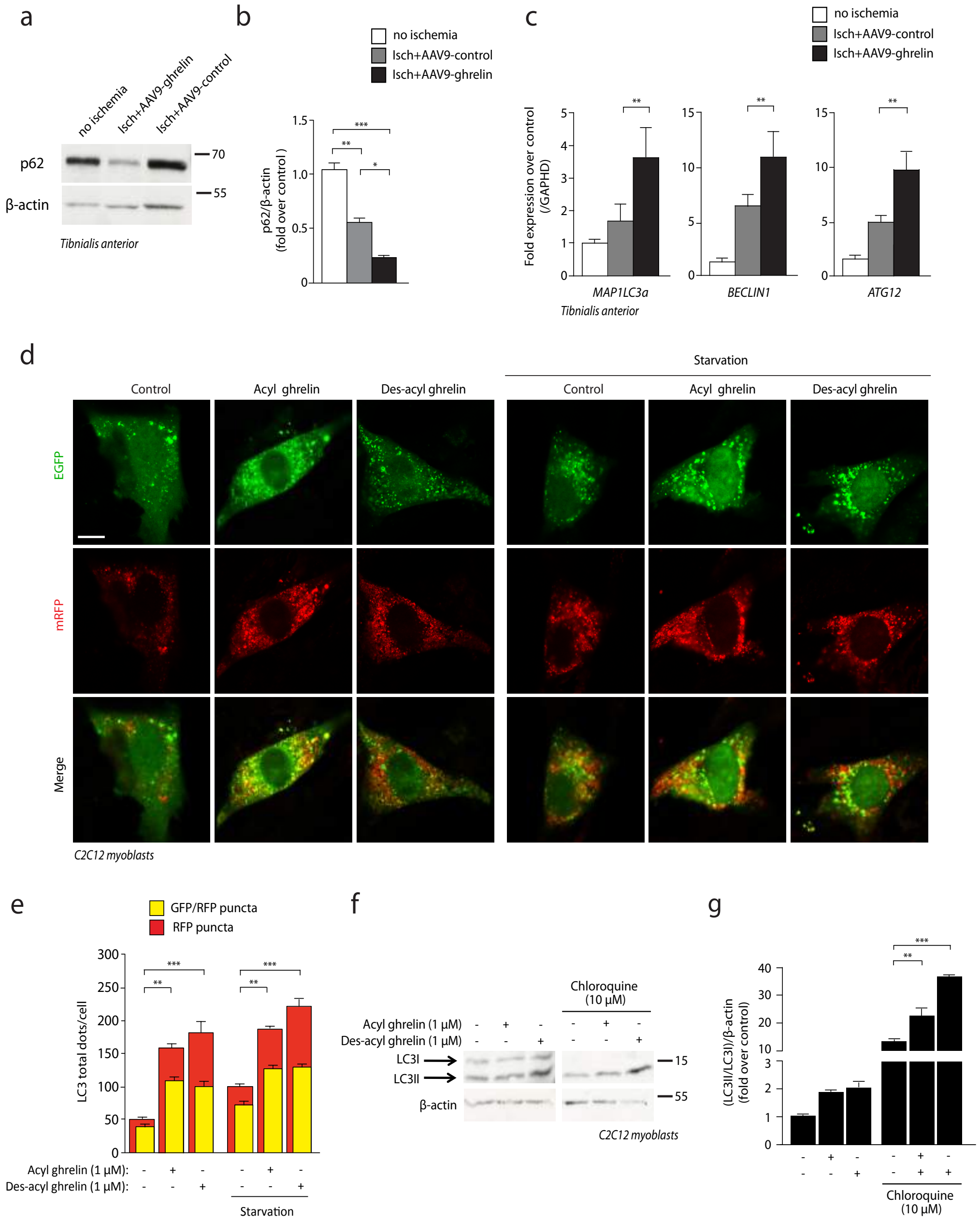

Supplementary Figure 6. Ghrelin stimulates autophagy after hind limb ischemia. (**a, b**) p62 protein levels in tibialis anterior muscle of control or ischemic mice harvested 7 days after peripheral ischemia. Representative western blot (**a**) and densitometric analysis (**b**; n=5). (**c**) Quantification of *MAP1LC3A*, *BECLIN1* and *ATG12* mRNA levels in tibialis anterior muscle of transduced mice 7 days after ischemia. Values are normalized for GAPDH and expressed as fold over untreated (n=5). (**d**) C2C12 cells transfected with the mRFP-EGFP tandem fluorescent-tagged LC3 plasmid (ptfLC3) and, after 48 hours, treated for 4 hours with acyl ghrelin, des-acyl ghrelin (both 1 μM) or vehicle in complete or starving medium. Scale bar=10 μm. (**e**) Quantification of EGFP and mRFP LC3 positive dots per cell, using the ImageJ software (number of analyzed cells: 30 per group). (**f, g**) LC3 lipidation in C2C12 myoblasts treated for 4 hours with acyl ghrelin, des-acyl ghrelin (both 1 μM) or vehicle in presence or absence of chloroquine (10 μM). Representative western blot (**f**) and densitometric analysis (**g**) of the ratio between LC3II and LC3I (n=3). All values are mean±SEM. One-way ANOVA and Bonferroni/Dunn's post hoc test were used to compare multiple groups (b, c, e, g) *$P<0.05$, **$P<0.01$, ***$P<0.001$ relative to control.

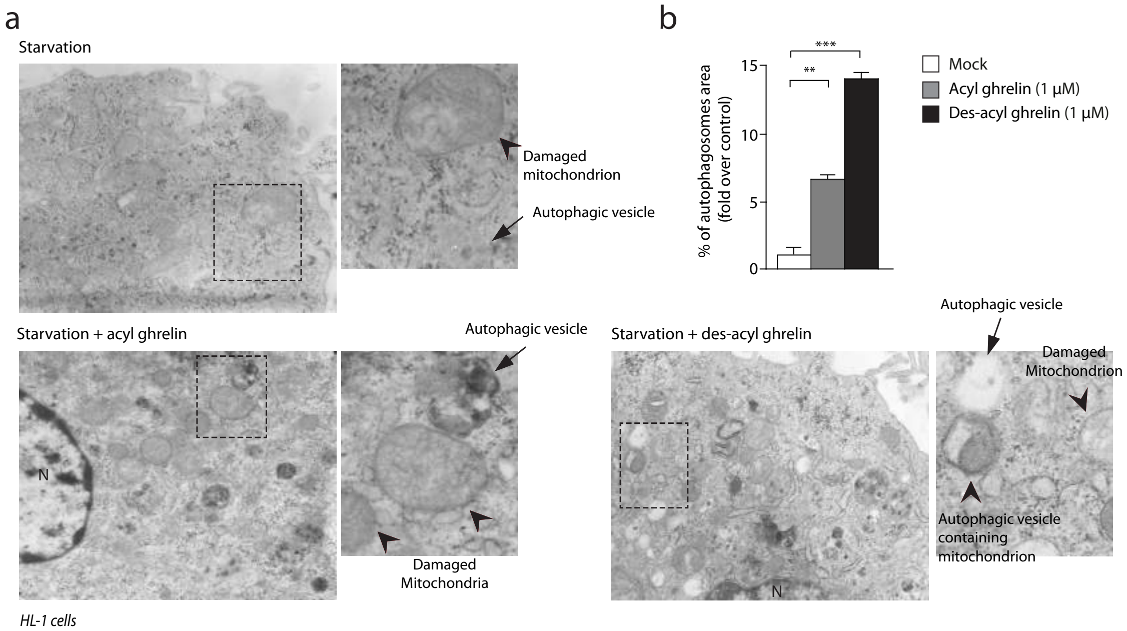

Supplementary Figure 7. Des-acyl ghrelin increases autophagy and the removal of damaged mitochondria after serum and glucose starvation. (**a**) Representative electron micrographs of HL-1 cells incubated in starving medium (-glucose, -serum) with acyl ghrelin, des-acyl ghrelin (both 1 µM) or vehicle for 4 hours. For each picture, an enlargement of the image is displayed, showing the presence of normal or damaged mitochondria (indicated by arrowheads) and autophagic vesicles (indicated by arrows). Scale bar=1 µm. (**b**) Quantification from the EM pictures of the percentage of autophagic structures area per cell section, calculated as relative fold over mock condition (n=10 per condition). Values are mean±SEM. One-way ANOVA and Bonferroni/Dunn's post hoc test were used to compare multiple groups $^{**}P<0.01$, $^{***}P<0.001$ relative to control.

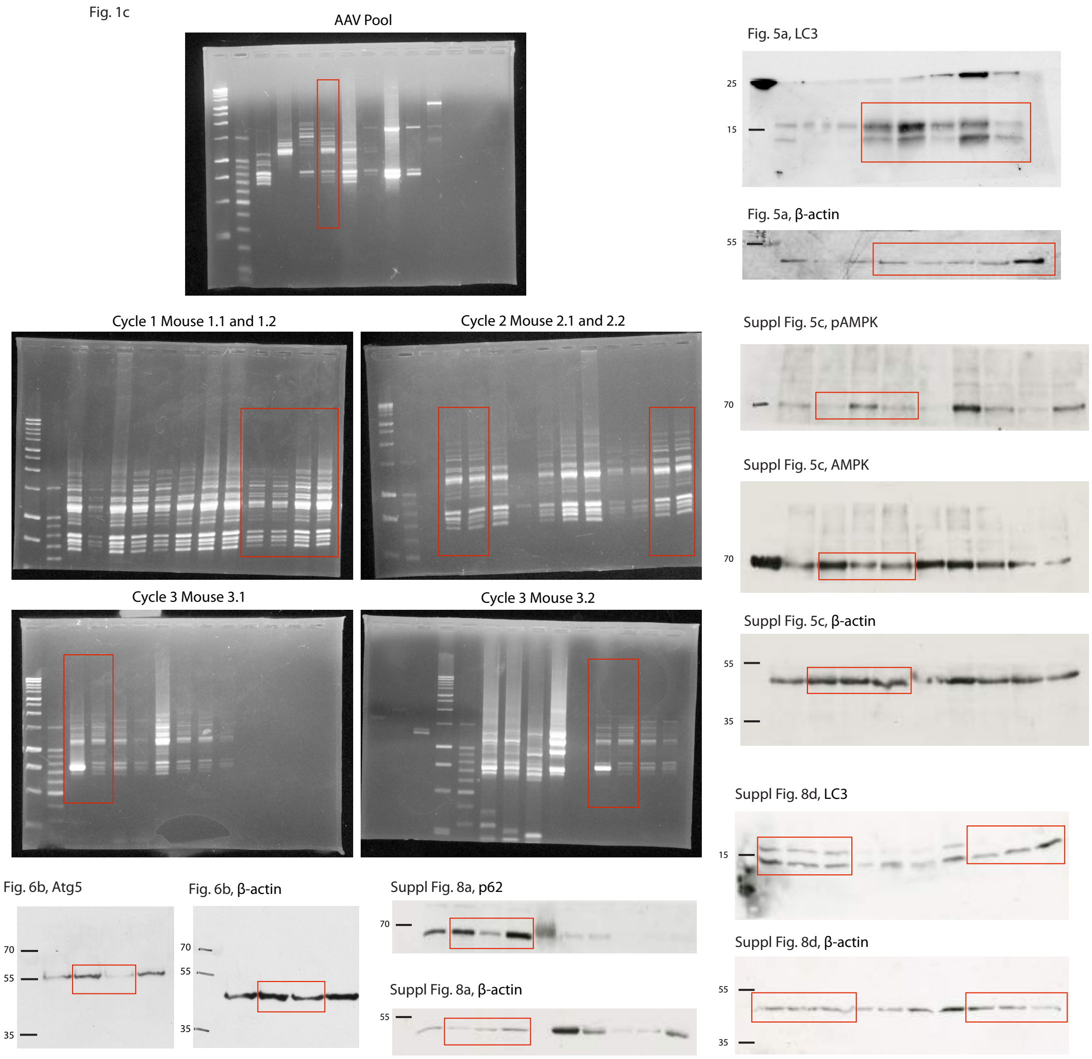

Supplementary Figure 8. Full-length uncropped images of gels displayed in the figures.

Supplementary Table 1. Parameters affecting FunSel performance

Variable	Description	Symbol	Range of values
Efficacy of transduction	Measures the fraction of transduced cells	ET	>0 to 1 (ET=1 all the cells are transduced)
Multiplicity of infection	Measures the number of AAV genomes per cell	MOI	>0
Efficiency of selection	Measures at what extent the transgene confers a selective advantage to the transduced cells	ES	>0 to 1 (ES=1 all the cells not containing the desired transgene are eliminated after selection)
Co-selection effect	Measures the extent of the transgene effect on neighboring, non-transduced cells	COSEL	0 or >0
Complexity of vector pool	How many different AAV vector are used for simultaneous transduction	POOL	>1
Number of selection cycles	How many cycles of iterative selection are carried out	CYCLE	1 or >1

Supplementary Table 2. List of factors in the AAV arrayed library

	Clone	Gene Name	Symbol	Gene ID	Pool 25	Pool 50	AAV9-Pool30
**Chemokines and Interleukins**	4A2	chemokine (C-C motif) ligand 6	Ccl6	20305	X	X	
	G4	chemokine (C-C motif) ligand 7	Ccl7	20306	X	X	X
	D8	chemokine (C-C motif) ligand 8	Ccl8	20307	X	X	X
	E4	chemokine (C-C motif) ligand 11	Ccl11	20292	X	X	X
	E10	chemokine (C-C motif) ligand 12	Ccl12	20293		X	X
	H2	chemokine (C-X-C motif) ligand 9	Cxcl9	17329	X	X	X
	F3	chemokine (C-X-C motif) ligand 13	Cxcl13	55985			
	G11	chemokine (C motif) ligand 1	Xcl1	16963			
	G12	interleukin17A	IL17A	16171	X	X	X
	G2	interleukin 20	IL20	58181		X	X
	B6	interleukin 25	IL25	140806			
**Hormones and Growth Factors**	H3	apelin	Apln	30878			
	4F7	family with sequence similarity 3, member B	Fam3b	52793		X	
	H5	family with sequence similarity 181, member B	Fam181b	58238			
	3D11	family with sequence similarity 198, member A	Fam198a	245050		X	
	2B11	follicle stimulating hormone beta	Fshb	14308			
	D10	gastric inhibitory polypeptide	Gip	14607			
	D11	gastrin	Gast	14459	X	X	X
	E2	ghrelin	Ghrl	58991	X	X	X
	2A10	olfactomedin-like 3	Olfml3	99543			
	C12	insulin-like 6	Insl6	27356	X	X	X
	G8	natriuretic peptide precursor type C	Nppc	18159	X	X	X
	F9	neuromedin U	Nmu	56183	X	X	X
	4D10	neurotensin	Nts	67405			
	F8	pro-opiomelanocortin-alpha	Pomc	18976	X	X	X
	3H10	peptidoglycan recognition protein1	Pglyrp1	21946			
	C10	prolactin family 3, subfamily b, member 1	Prl3b1	18776	X	X	X
	B10	prolactin family 3, subfamily d, member 1	Prl3d1	18775	X	X	X
	C7	prolactin family 5, subfamily a, member 1	Prl5a1	28078			X
	C8	prolactin family 7, subfamily b, member 1	Prl7b1	75596			X
	F1	prolactin family 7, subfamily d, member 1	Prl7d1	18814	X	X	X
	3F5	prolactin family 8, subfamily a, member 81	Prl8a8	74188	X	X	
	1F4	prolactin family8, subfamily a, member 9	Prl8a9	67310			
	4A11	trefoil factor 1	Tff1	21784		X	
	C5	Trophoblast specific protein beta	Tpbpb	116913			X
	E7	thyrotropinreleasinghormone	Trh	22044			
	G5	vasoactive intestinal polypeptide	Vip	22353	X	X	X
	3C7	amylase 1, salivary	Amy 1	11722		X	
	3C2	arylsulfatase A	Arsa	11883			

**Secreted Enzymes**	1F6	biotinidase	Btd	26363			
	2C12	carboxylesterase 2A	Ces2a	102022		X	
	3D10	mannan-binding lectin serine protease 2	Masp2	17175		X	
	1D10	chitinase-like 1	Chil1	12654			
	1G3	colipase	Clps	109791		X	X
	3G4	cystatin 9	Cst9	13013			
	4F12	cystatin 11	Cst11	78240			
	D1	cystatin 12	Cst12	69362		X	X
	3F6	cystatin 13	Cst13	69294		X	
	4A5	chymotrypsinogen B1	Ctrb1	66473			
	1C10	glucosidase, beta, acid	Gba	14466			
	H9	kallikrein 1	Klk1	16612		X	
	3G6	lysozyme-like 1	Lyzl1	67328			
	3D6	matrix metallopeptidase 1a	Mmp1a	83995		X	
	3A6	napsin A aspartic peptidase	Napsa	16541			
	4B1	progastricsin (pepsinogen C)	Pgc	109820		X	
	4B2	phospholipase A2, group IB	Pla2g1b	18778		X	
	3F12	protease, serine, 37	Prss37	67690			
	3G7	protease-associated domain containing 1	Pradc1	73327		X	
	1E2	renin 1	Ren1	19701		X	X
**Extracellular Matrix Proteins**	1C6	amelogenin X chromosome	Amelx	11704			
	1E8	asporin	Aspn	66695			
	1C8	epiphycan	Epyc	13516			
	4F1	fibroomodulin	Fmod	14264			
	B11	fibulin 1	Fbln1	14114			
	4H6	glypican 5	Gpc5	103978			
	D5	intelectin b	Itlnb	493583			X
	3E4	keratocan	Kera	16545	X	X	
	F7	lumican	Lum	17022			
	3D2	matrilin 3	Matn3	17182	X	X	
	H10	matrix metallopeptidase 12	Mmp12	17381			
	4E8	nephrocan	Nepn	66650			
	1D6	nephronectin	Npnt	114249	X	X	
	4C10	spondin 2	Spon2	100689			
	1D12	transforming growth factor, beta induced	Tgfbi	21810	X	X	
	H12	vitronectin	Vtn	22370	X	X	X
**Secreted Molecules of unknown function**	G7	RIKEN cDNA D730048I06 gene	D730048I06Rik	68171		X	X
	3G12	RIKEN cDNA 1810009J06 gene	1810009J06Rik	73626			
	F6	RIKEN cDNA 5530400C23 gene	5530400C23Rik	232426			X
	3G5	RIKEN cDNA 1700029I15 gene	1700029I15Rik	75641			
	3H9	RIKEN cDNA 2010109I03 gene	2010109I03Rik	67038	X	X	
	4G10	RIKEN cDNA 9530003J23 gene	9530003J23Rik	77397			
	3D1	RIKEN cDNA 4930568D16 gene	4930568D16Ri	75859			
	B7	RIKEN cDNA 4930597L12 gene	4930597L12Rik	75385		X	

	4E1	RIKEN cDNA 5430402E10 gene	5430402E10Rik	71351			
	2D2	WAP four disulfide core domain 15B	Wfdc15b	192201			
**Others**	F2	alpha fetoprotein	Afp	11576	X	X	
	3E6	anterior gradient homolog 3	Agr3	403205		X	
	3G9	BPI fold containing family A, member 3	Bpifa3	73388			
	2F8	coagulation factor VII	F7	14068			
	1D3	complement component 8, beta polypeptide	C8b	110382		X	
	G6	defensin beta 20	Defb20	319579			
	4G2	lipocalin 9	Lcn9	77704		X	
	4E6	odorant binding protein1a	Obp1a	18249			
	G1	oocyte secreted protein 1	Oosp1	170834			X
	3C5	proacrosin binding protein	Acrbp	54137		X	
	D7	regenerating islet-derived 3 delta	Reg3d	30053	X	X	X
	4A4	regenerating islet-derived 3 gamma	Reg3g	19695		X	
	3G1	salivary protein 1	Spt1	20770			
	1H5	sclerostin domain containing 1	Sostdc1	66042			
	4C4	wingless-related MMTV integration site 2	Wnt2	22413		X	

Supplementary Table 3. Primers and probes

**Primers for cDNAs subcloning into the pZac2.1 AAV-backbone plasmid**	
pBluescript FW	5'-CTGTCCGCTCGAGCCAGTGAATTGTAATACGAT-3'
pFlcI FW	5'-CTGTCCGCTCGAGTACGAAGTTATGGATCAGGC-3'
pB/pF RV (common)	5'-ATAAGAATGCGGCCGCCTAAAGGGAACAAAAGC-3'
**Primers for AAV insert amplification**	
pZac2.1 FW	5'-GTGTCCACTCCCAGTTCAAT-3'
pZac2.1 RV	5'-GTGGTTTGTCCAAACTCATC-3'
**Primers and probes for viral DNA quantification**	
Mouse D11 (*GAST*)	F: 5’-GTGGACAAGATGCCTCGACT-3’<br>R: 5’-TGTTCCAGGTCCTCATTGGT-3’
Mouse E2 (*GHRL*)	F: 5’-GCCCAGAGCACCAGAAAG-3’<br>R: 5’-TGATCTCCAGCTCCTCCTCT-3’
Mouse C12 (*INSL6*)	F: 5’-AACCCTCACCCTTCTTCCTC-3’<br>R: 5’-GCTCCCAAGTGTGTGTTCCT-3’
Mouse G4 (*CCL7*)	F: 5’-CCCAAGAGGAATCTCAAGAGC-3’<br>R: 5’-AGCCTCCTCGACCCACTTCT-3’
Mouse F8 (*POMC*)	F: 5’-CACTGAACATCTTTGTCCCCA-3’<br>R: 5’-CGACTGTAGCAGAATCTCGG-3’
Mouse H2 (*CXCL9*)	F: 5’-CCGCTGTTCTTTTCCTCTTG-3’<br>R: 5’-GCATCGTGCATTCCTTATCA-3’
Mouse G5 (*VIP*)	F: 5’-TTCACCAGCGATTACAGCAG-3’<br>R: 5’-GTGTCGTTTGATTGGCACAG-3’
Mouse H12 (*VTN*)	F: 5’- GTACTGGCGCTTTGAGGATG-3’<br>R: 5’- AACGCTGCATCAACATTGTC-3’
Mouse F2 (*AFP*)	F: 5’-CCAAAGCATTGCACGAAAAT-3’<br>R: 5’-CTTCCGGAACAAACTGGGTA-3’
Mouse 1D12 (*TGFBI*)	F: 5’-ATCACCAACAACATCCAGCA-3’<br>R: 5’-CCAGCACGGTATTGAGTCCT-3’
Mouse 1F6 (*BTD*)	F: 5’-ATCAGCAGGAGGCGGGACGAG-3’<br>R: 5’-AGCGCAGAACAGCCGAGGAA-3’

Mouse 2C12 (*CES2A*)	F: 5’-GCCTGACCTCATCTCTGACACTT-3’<br>R: 5’-ACTCTTTGCTCTCAGGCAGTGGA-3’
Mouse 1C10 (*GBA*)	F: 5’- CCTGCATCCCCAAAAGCTTT-3’<br>R: 5’- GTAAGGTCACGGGGTCAAGA-3’
Mouse 4C10 (*SPON2*)	F: 5’-ACTCGCTGGTGTCCTTCGTGGT-3’<br>R: 5’-TCGGTCCCTGCATCGTGTG-3’
Mouse 1D3 (*C8B*)	F: 5’-ACCAAACGCTTCGCCCACACT-3’<br>R: 5’-GCAGGCTCTTGACCCTCTGA-3’
Mouse 1D6 (*NPNT*)	F: 5’- AAGTGCCCTATCGTGTTCCA-3’<br>R: 5’- CTCTTCCAGTCGCACATTCA-3’
Mouse 3D10 (*MASP2*)	F: 5’- CCTCCCAGGATTGAAACTGA-3’<br>R: 5’- CCGTGCTGTGCTTGTGTAGT-3’
CMV DNA	F: 5’-TGGGCGGTAGGCGTGTA-3’<br>R: 5’-GATCTGACGGTTCACTAAACGAG-3’<br>Taqman Probe: (FAM)-TGGGAGGTCTATATAAGC
**Primers for cellular RNA quantification by SYBRGreen**	
Mouse *IL-1β*	F: 5’-GCTGTGGCAGCTACCTGTGTCTT-3’<br>R: 5’-GGGAACGTCACACACCAGCAGGT-3’
Mouse *TNFα*	F: 5’-CCACCACGCTCTTCTGTCTAC-3’<br>R: 5’-TTGGGAACTTCTCATCCCTTT-3’
Mouse *β-MHC*	F: 5’-TCCGCAAGGTGCAGCACGAG-3’<br>R: 5’-CACGGGCACCCTTGGAGCTG-3’
Mouse *α-MHC*	F: 5’-ATGTTAAGGCCAAGGTCGTG-3’<br>R: 5’-CACCTGGTCCTCCTTTATGG-3’
Mouse *SERCA2a*	F: 5’-CGCTACGGTGCCTGGCTCTG-3’<br>R: 5’-TGCCCACACAGCCGACGAAA-3’
Mouse *RYR2*	F: 5’-AAGGCGAGGATGAGATCCAGTTC-3’<br>R: 5’-ATCCTTCTGCTGCCAAGCACAGCT-3’
Mouse *NPPA*	F: 5’-TCCAGCTGCTTCGGGGGTAGG-3’<br>R: 5’-CCGCAGCTCCAGGAGGGTGT-3’
Mouse *BNP*	F: 5’-GTCCTTCGGTCTCAAGGCAGCAC-3’<br>R: 5’-GGGAAAGAGACCCAGGCAGAGTC-3’
Mouse *PGC1α*	F: 5’-CAACCGCAGTCGCAACATGCTCA-3’<br>R: 5’-CTTGGGGTCATTTGGTGACTCTG-3’

**Taqman probes (assay) for RNA quantification**	
Mouse *GHRL*	Mm00445450_m1
Mouse *IL6*	Mm00446190_m1
Mouse *BAX*	Mm00432051_m1
Mouse *BCL-2*	Mm00477631_m1
Mouse *MAP1LC3a*	Mm00458725_g1
Mouse *BECLIN1*	Mm01265461_m1
Mouse *ATG12*	Mm00503201_m1
